# Supplementary material for: Somatostatin triggers local cAMP and Ca2+ signaling in primary cilia to modulate pancreatic β-cell function
Source: EMBO J. 2025 Feb 12;44(6):1663–91. doi: 10.1038/s44318-025-00383-7 (PMC11914567; doi:10.1038/s44318-025-00383-7)
Supplement: Supplementary file 1 — Appendix [file 44318_2025_383_MOESM1_ESM.pdf]

APPENDIX

Table of contents

Appendix figure S1

Appendix figure S2

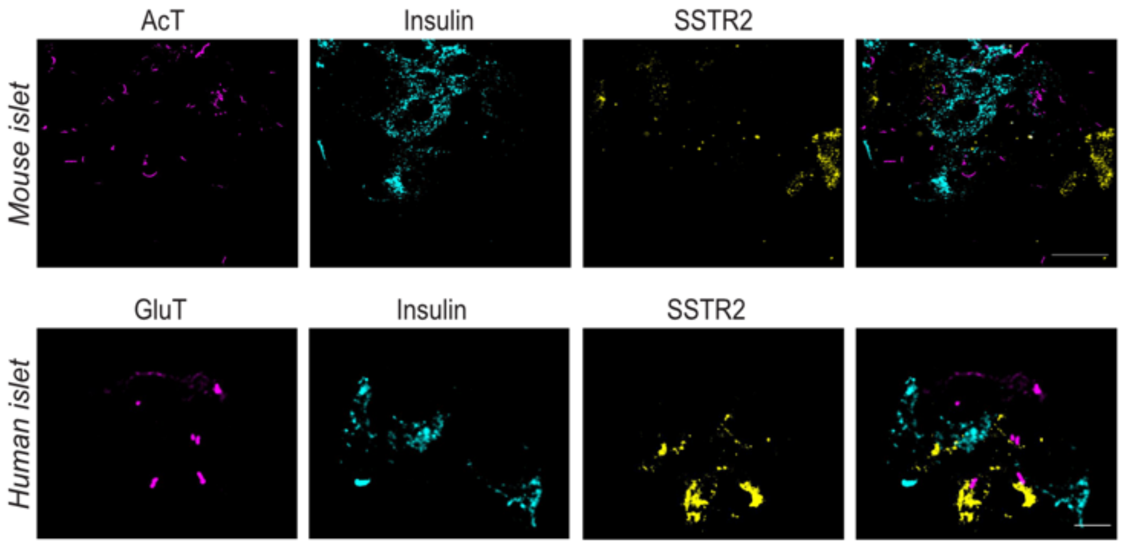

Appendix figure S1. Localization of SSTR2 in mouse and human islets.
Confocal microscopy images of a mouse and human islet immunostained against SSTR2 (yellow), acetylated tubulin and glutamylated tubulin as cilia markers (magenta) and insulin (cyan).

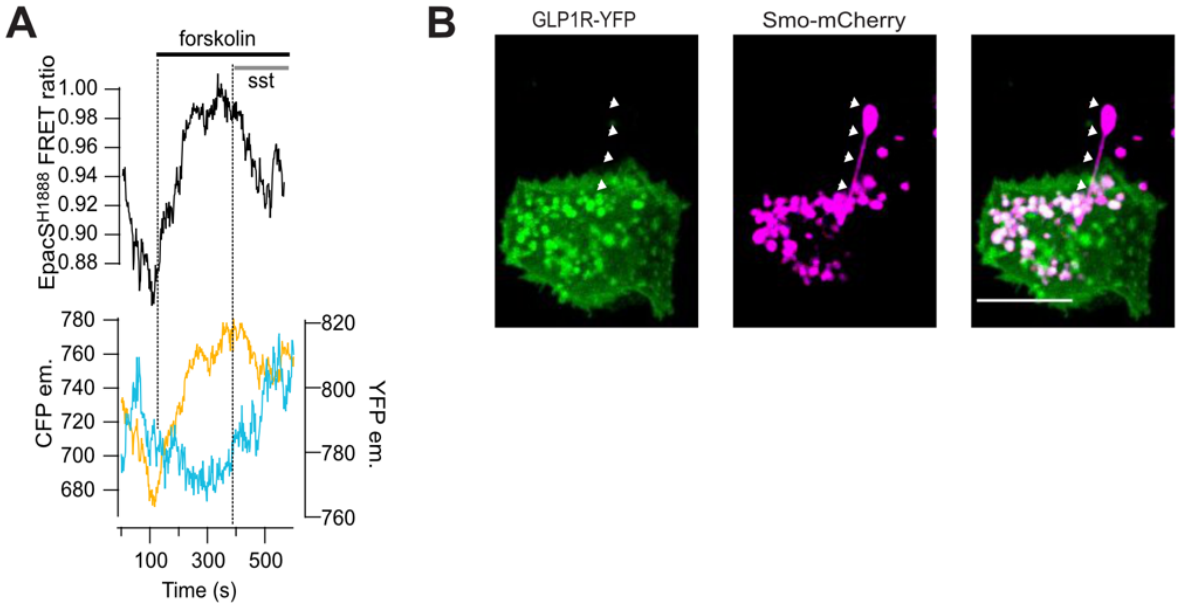

Apendix figure S2. FRET based detection of ciliary cAMP.
A. Representative recording of mArl13b-188 FRET ratio change from a MIN6 cell in response to 10 µM forskolin and 100 nM somatostatin (sst).
B. Representative confocal microscopy images showing non-ciliary localization of GLP-1R (green) in MIN6 cells and mouse islets. MIN6 cells overexpress GLP-1R-YFP (green) and Smo-mCherry (magenta).
