# Supplementary figures and images for: Somatostatin triggers local cAMP and Ca2+ signaling in primary cilia to modulate pancreatic β-cell function (part 1 of 2)
Source: EMBO J. 2025 Feb 12;44(6):1663–91. doi: 10.1038/s44318-025-00383-7 (PMC11914567; doi:10.1038/s44318-025-00383-7)

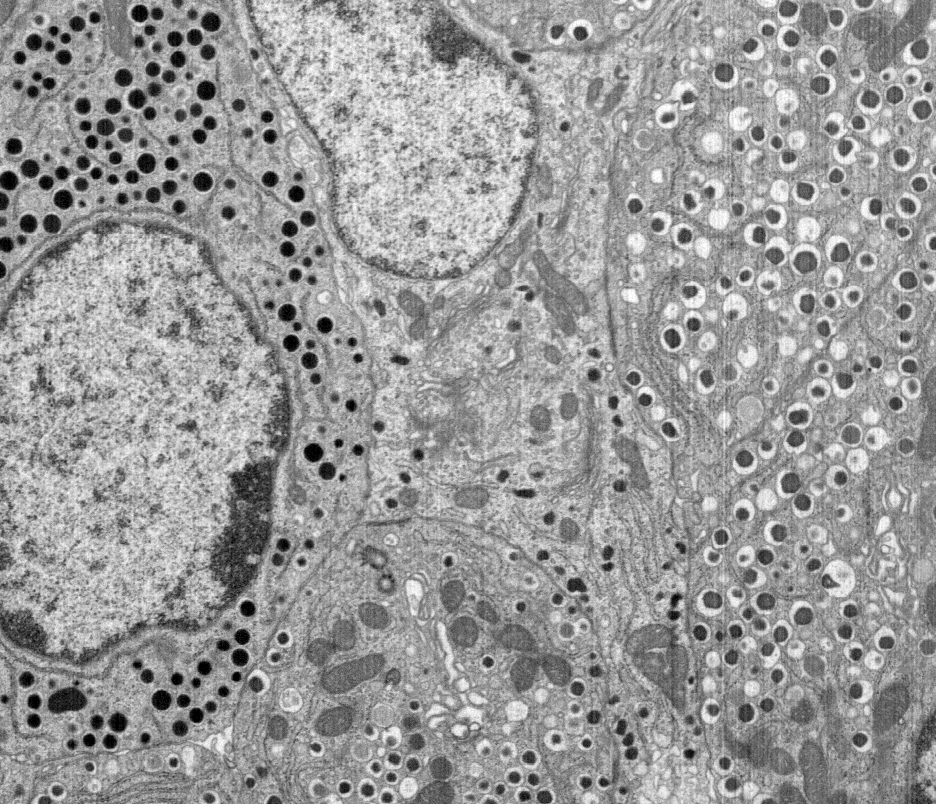

Supplement: Supplementary file 3 — Source data Fig. 1 [file 44318_2025_383_MOESM3_ESM.zip › Figure 1/1A/Fig1A-1.tif]

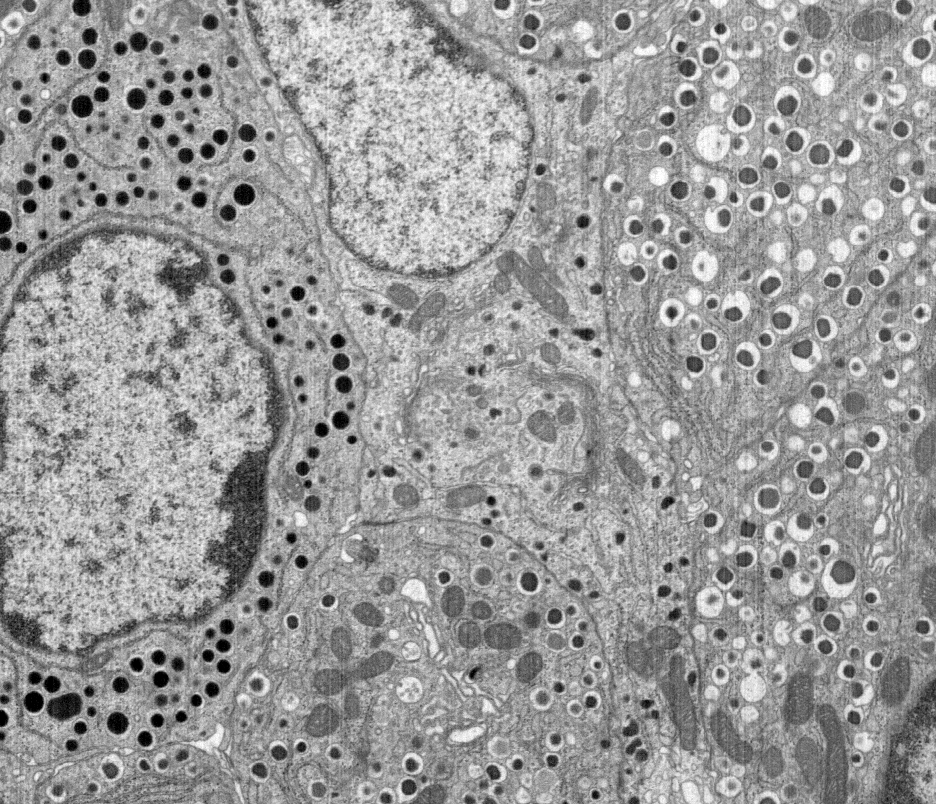

Supplement: Supplementary file 3 — Source data Fig. 1 [file 44318_2025_383_MOESM3_ESM.zip › Figure 1/1C/Fig1C-2.tif]

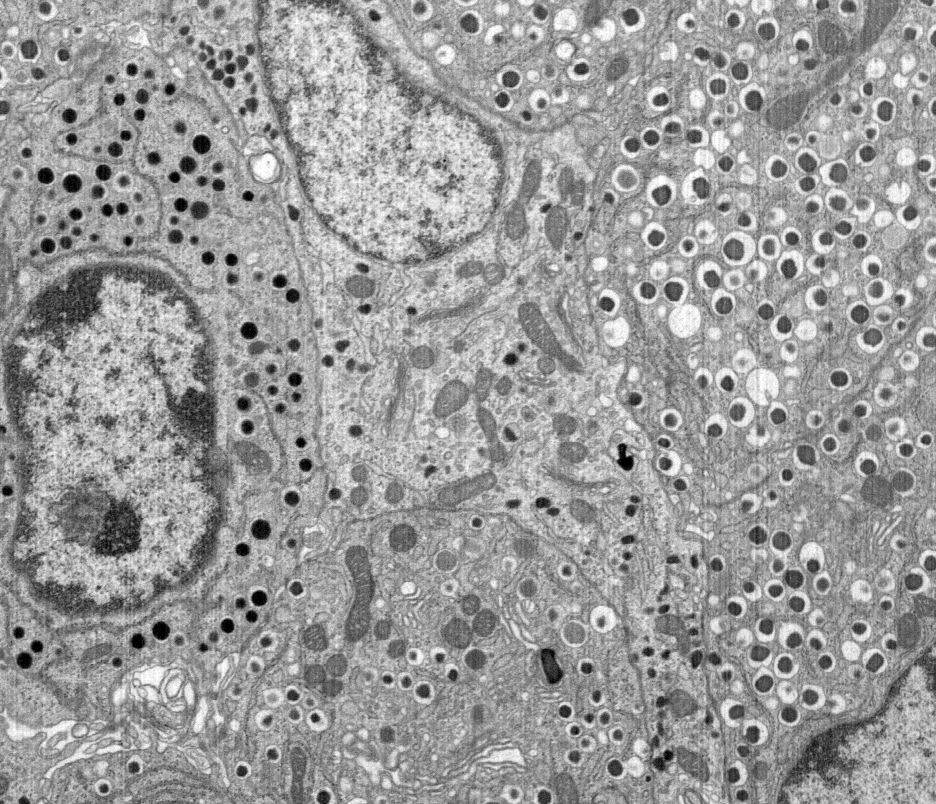

Supplement: Supplementary file 3 — Source data Fig. 1 [file 44318_2025_383_MOESM3_ESM.zip › Figure 1/1C/Fig1C-3.tif]

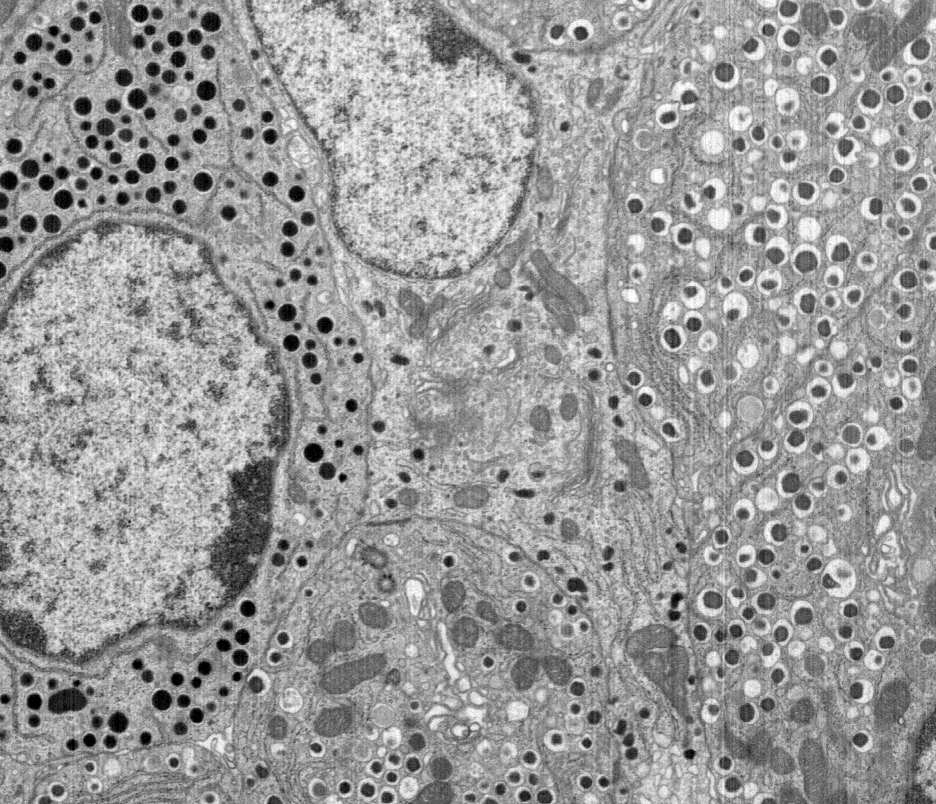

Supplement: Supplementary file 3 — Source data Fig. 1 [file 44318_2025_383_MOESM3_ESM.zip › Figure 1/1C/Fig1C-1.tif]

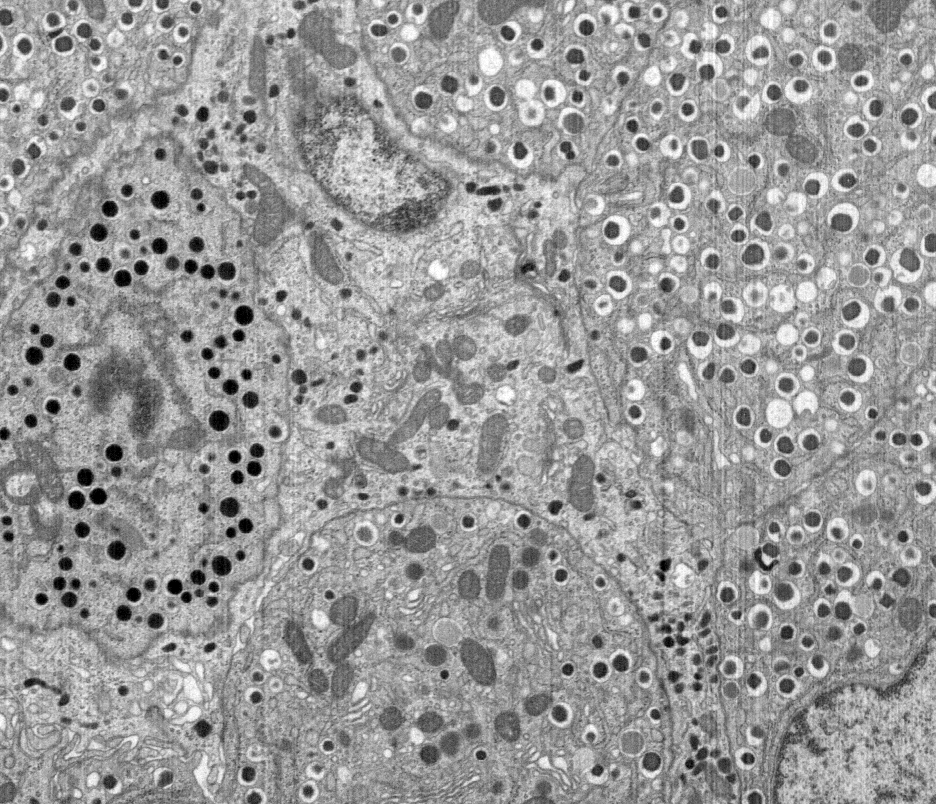

Supplement: Supplementary file 3 — Source data Fig. 1 [file 44318_2025_383_MOESM3_ESM.zip › Figure 1/1C/Fig1C-4.tif]

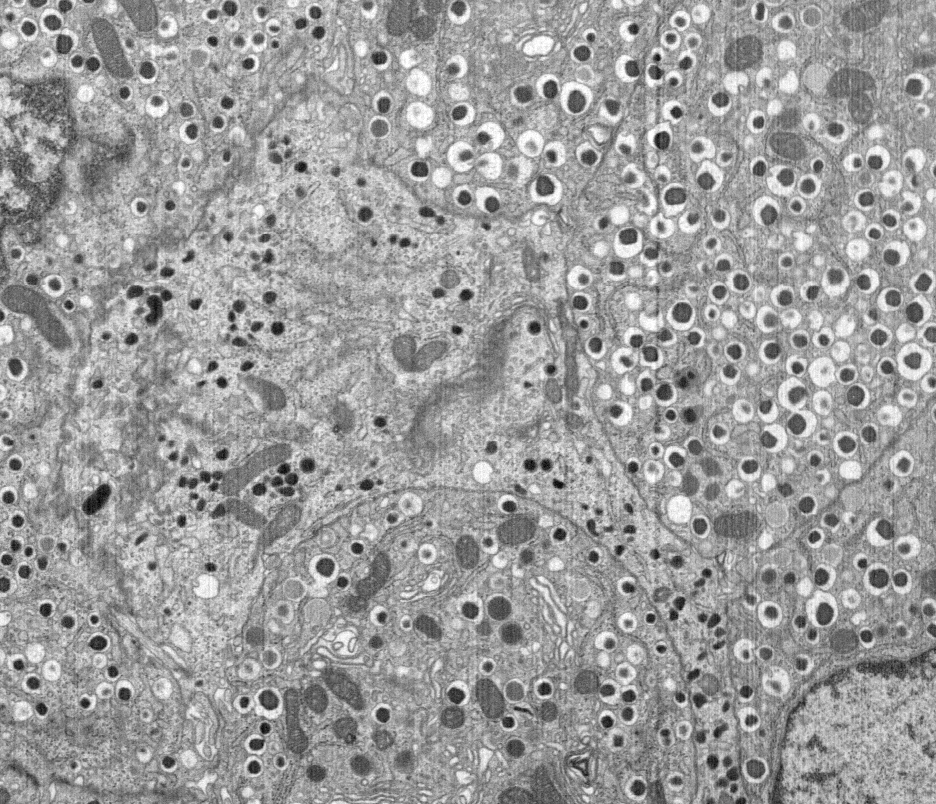

Supplement: Supplementary file 3 — Source data Fig. 1 [file 44318_2025_383_MOESM3_ESM.zip › Figure 1/1C/Fig1C-5.tif]

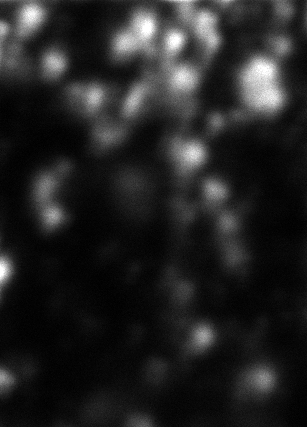

Supplement: Supplementary file 4 — Source data Fig. 2 [file 44318_2025_383_MOESM4_ESM.zip › Figure 2/Fig 2F/IMG0004_Mouse islets 18h SST STED.tif]

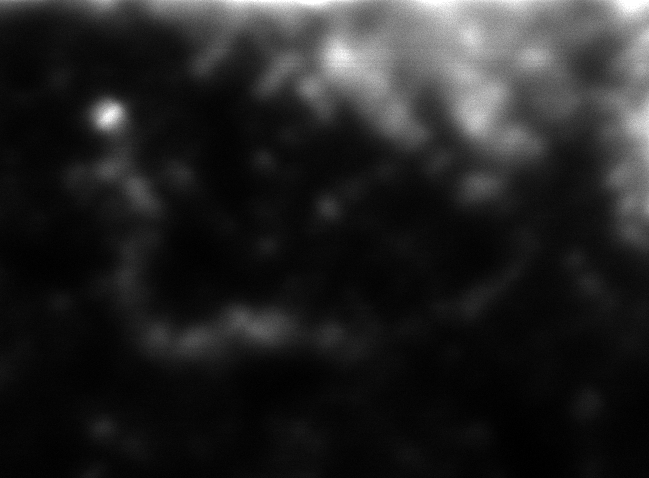

Supplement: Supplementary file 4 — Source data Fig. 2 [file 44318_2025_383_MOESM4_ESM.zip › Figure 2/Fig 2F/mouse islet SSTR3IMG0012_Control_STED.tif]

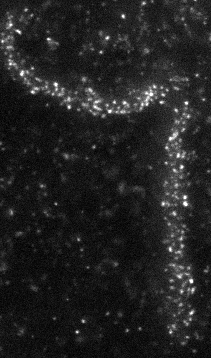

Supplement: Supplementary file 4 — Source data Fig. 2 [file 44318_2025_383_MOESM4_ESM.zip › Figure 2/Fig 2F/Figure2G 15 min SST.tif]

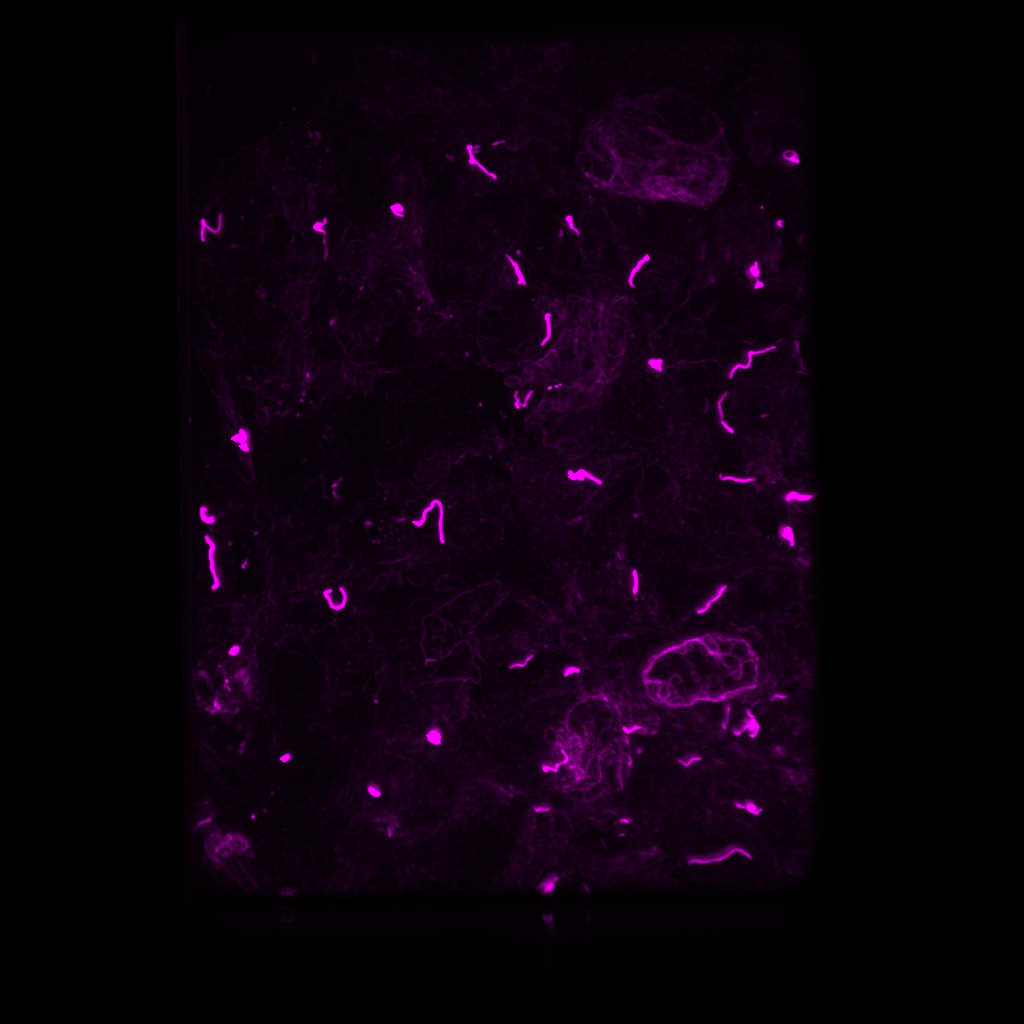

Supplement: Supplementary file 4 — Source data Fig. 2 [file 44318_2025_383_MOESM4_ESM.zip › Figure 2/2A/Actub.tif]

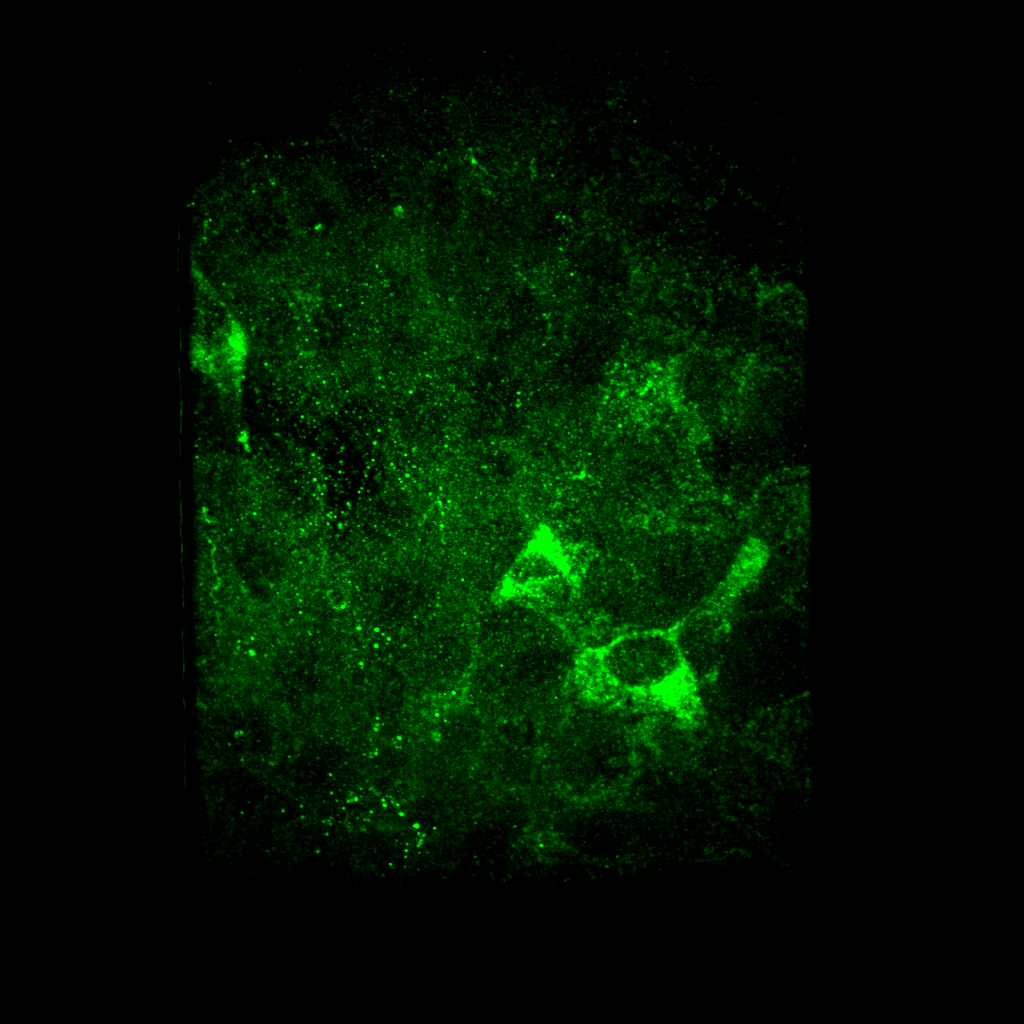

Supplement: Supplementary file 4 — Source data Fig. 2 [file 44318_2025_383_MOESM4_ESM.zip › Figure 2/2A/SSTR3.tif]

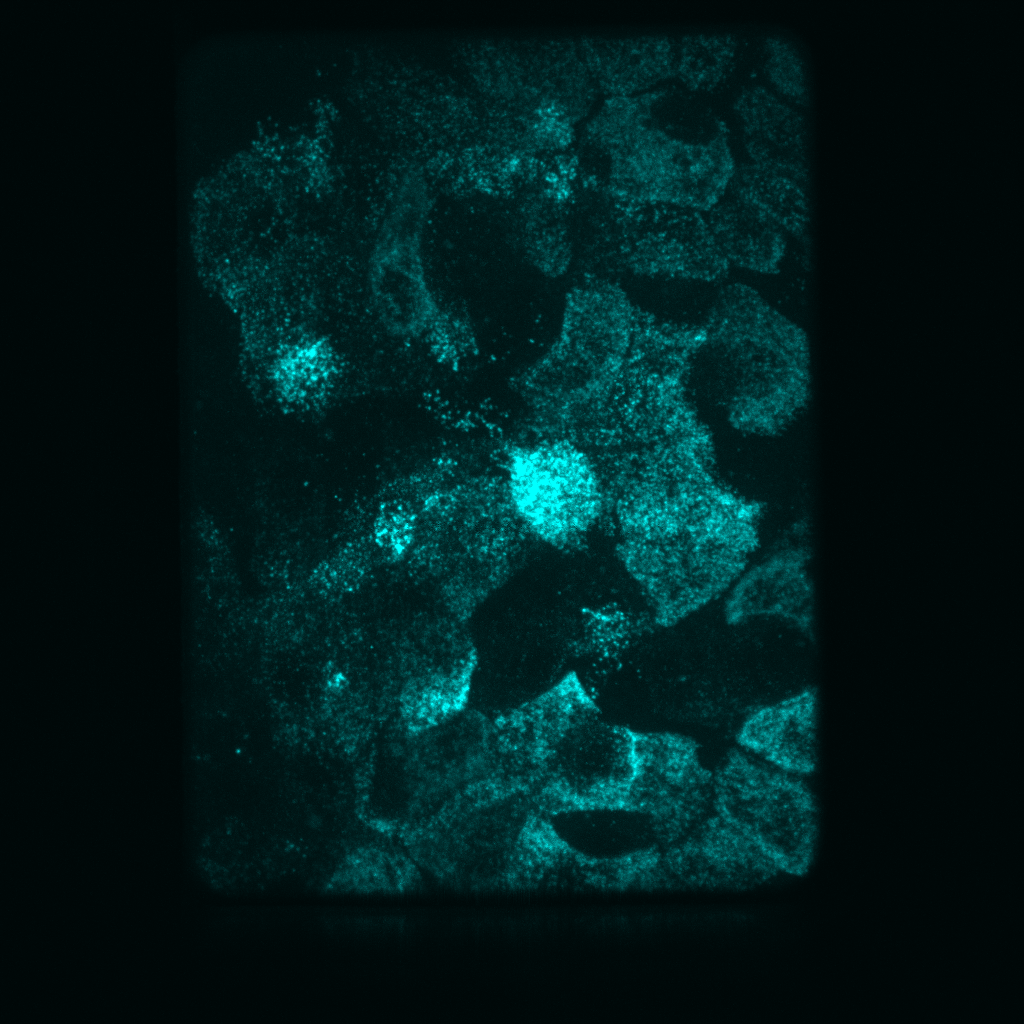

Supplement: Supplementary file 4 — Source data Fig. 2 [file 44318_2025_383_MOESM4_ESM.zip › Figure 2/2A/Insulin.tif]

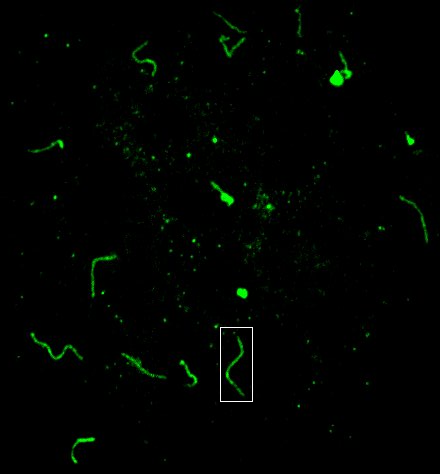

Supplement: Supplementary file 4 — Source data Fig. 2 [file 44318_2025_383_MOESM4_ESM.zip › Figure 2/2B/SSTR3_full.tif]

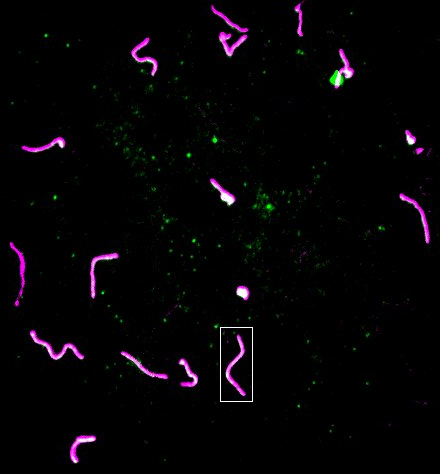

Supplement: Supplementary file 4 — Source data Fig. 2 [file 44318_2025_383_MOESM4_ESM.zip › Figure 2/2B/Composite_full.tif]

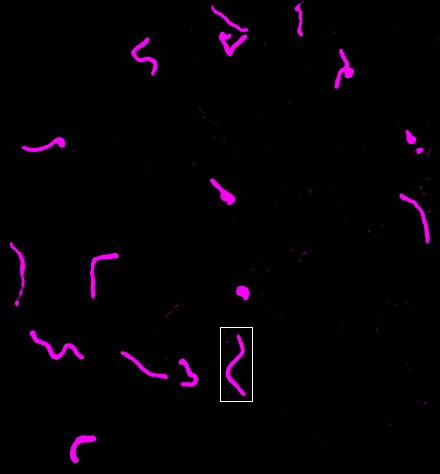

Supplement: Supplementary file 4 — Source data Fig. 2 [file 44318_2025_383_MOESM4_ESM.zip › Figure 2/2B/AcTub_full.tif]

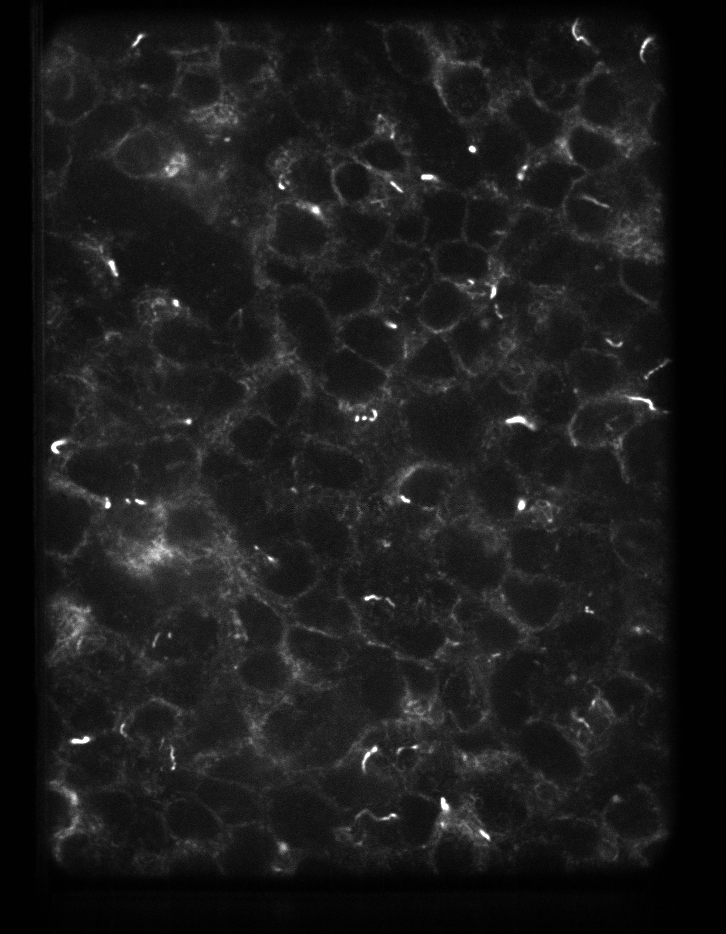

Supplement: Supplementary file 4 — Source data Fig. 2 [file 44318_2025_383_MOESM4_ESM.zip › Figure 2/2B/minPI SSTR3/min6PI staining.tif]

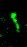

Supplement: Supplementary file 4 — Source data Fig. 2 [file 44318_2025_383_MOESM4_ESM.zip › Figure 2/2B/minPI SSTR3/SSTR3-2.jpg]

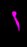

Supplement: Supplementary file 4 — Source data Fig. 2 [file 44318_2025_383_MOESM4_ESM.zip › Figure 2/2B/minPI SSTR3/AcT-2.jpg]

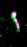

Supplement: Supplementary file 4 — Source data Fig. 2 [file 44318_2025_383_MOESM4_ESM.zip › Figure 2/2B/minPI SSTR3/merged-2.jpg]

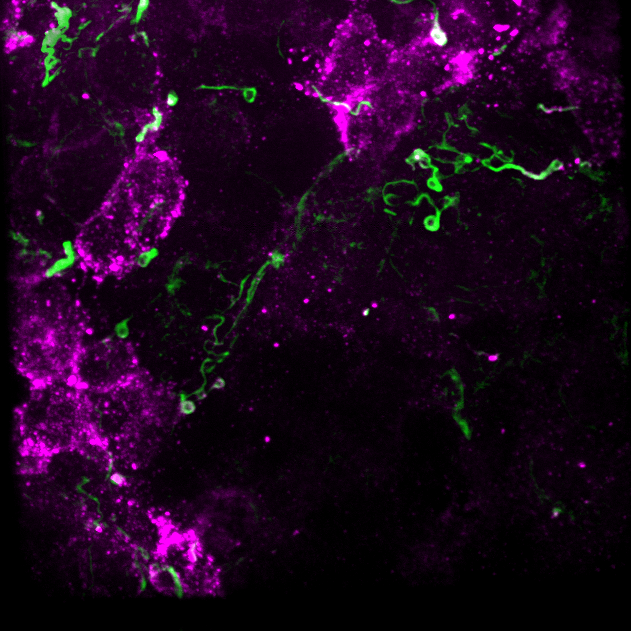

Supplement: Supplementary file 5 — Source data Fig. 3 [file 44318_2025_383_MOESM5_ESM.zip › Figure 3/3J/non-diabetic/Merge-1.tif]

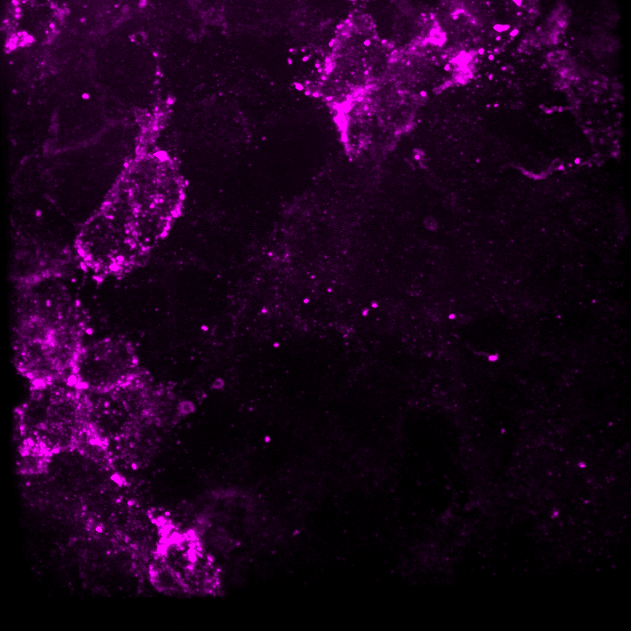

Supplement: Supplementary file 5 — Source data Fig. 3 [file 44318_2025_383_MOESM5_ESM.zip › Figure 3/3J/non-diabetic/SST-1.tif]

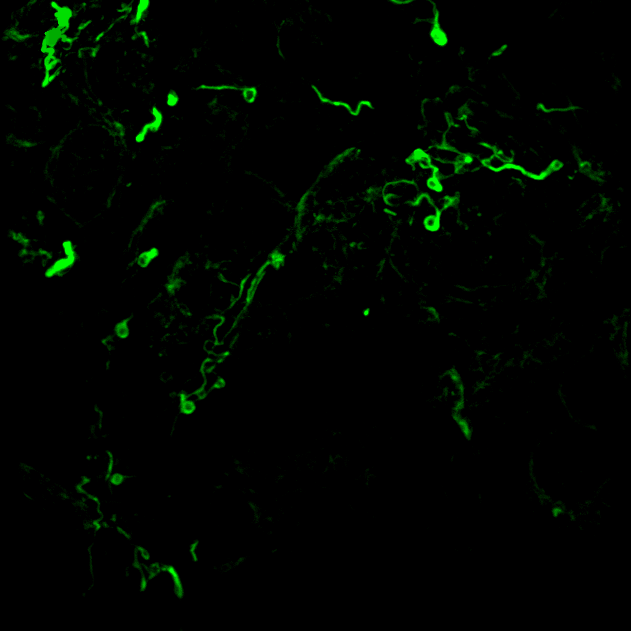

Supplement: Supplementary file 5 — Source data Fig. 3 [file 44318_2025_383_MOESM5_ESM.zip › Figure 3/3J/non-diabetic/cilia-1.tif]

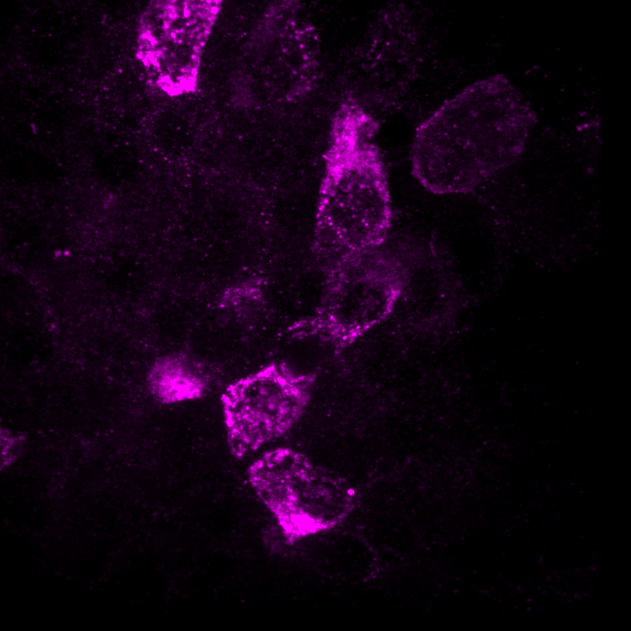

Supplement: Supplementary file 5 — Source data Fig. 3 [file 44318_2025_383_MOESM5_ESM.zip › Figure 3/3J/T2D/SST-T2D.tif]

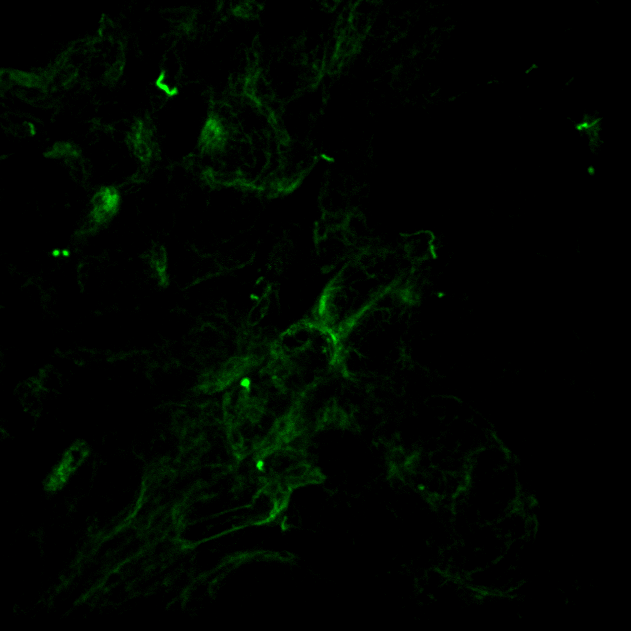

Supplement: Supplementary file 5 — Source data Fig. 3 [file 44318_2025_383_MOESM5_ESM.zip › Figure 3/3J/T2D/AcTub-T2D.tif]

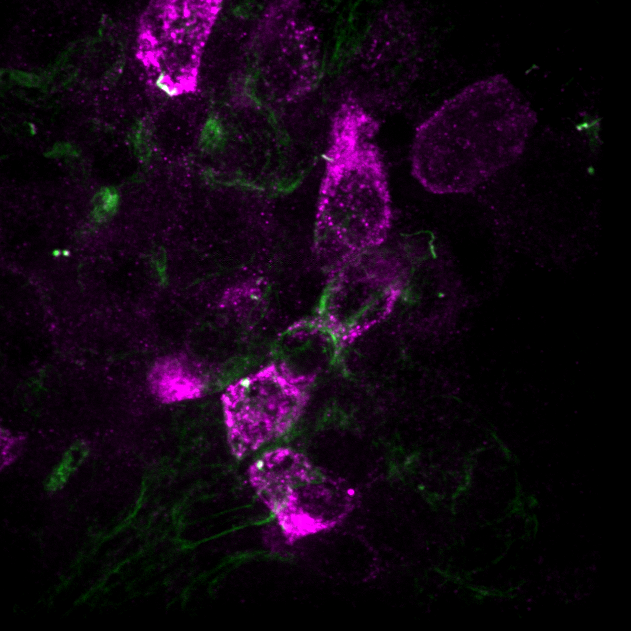

Supplement: Supplementary file 5 — Source data Fig. 3 [file 44318_2025_383_MOESM5_ESM.zip › Figure 3/3J/T2D/Composite-T2D.tif]

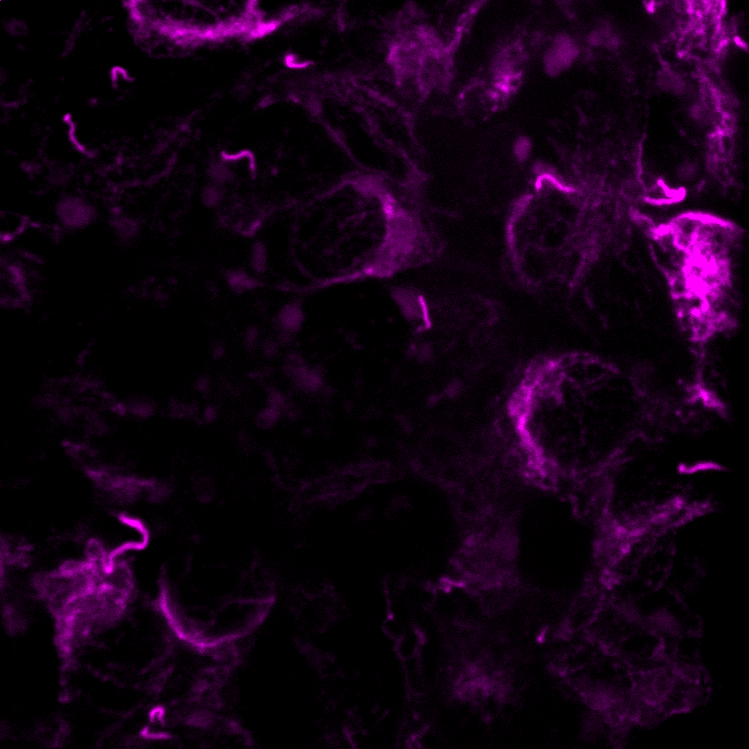

Supplement: Supplementary file 5 — Source data Fig. 3 [file 44318_2025_383_MOESM5_ESM.zip › Figure 3/3A/ND/AcT.tif]

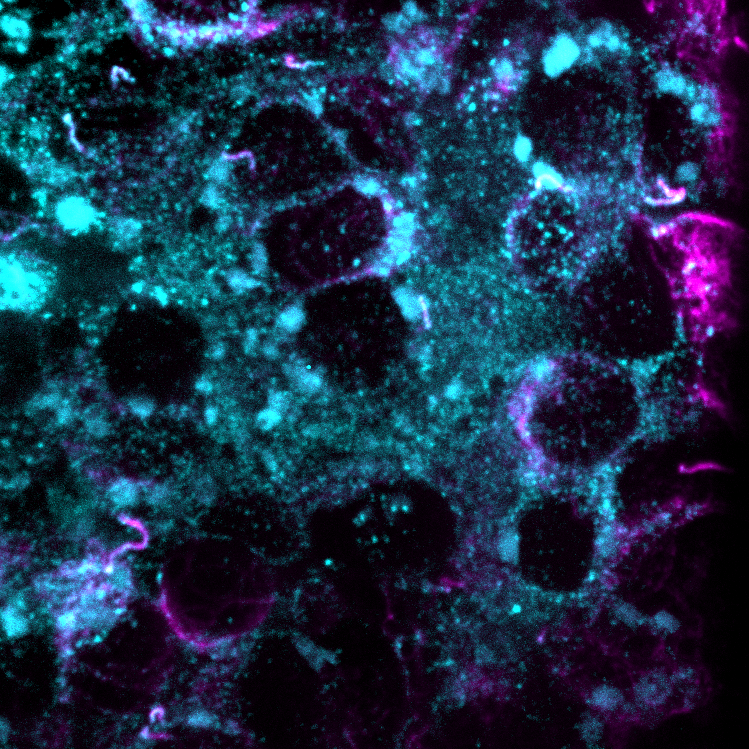

Supplement: Supplementary file 5 — Source data Fig. 3 [file 44318_2025_383_MOESM5_ESM.zip › Figure 3/3A/ND/MAX_Composite-4.tif (RGB).tif]

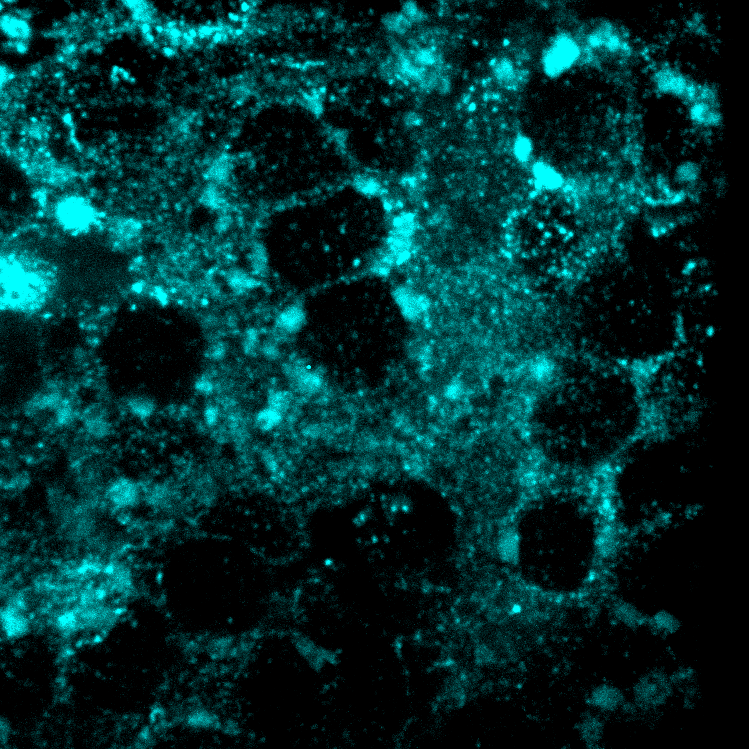

Supplement: Supplementary file 5 — Source data Fig. 3 [file 44318_2025_383_MOESM5_ESM.zip › Figure 3/3A/ND/Ins.tif]

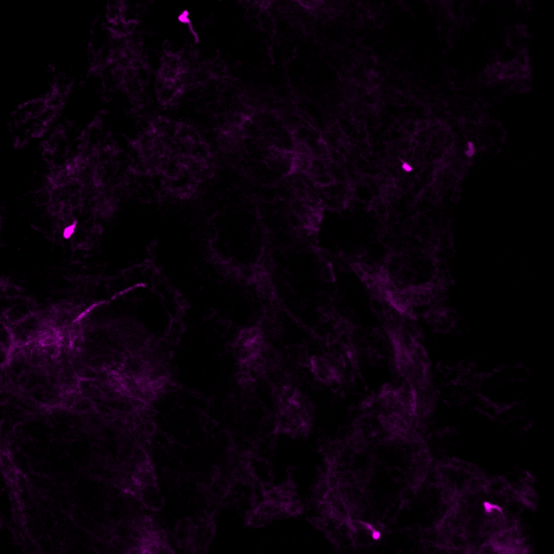

Supplement: Supplementary file 5 — Source data Fig. 3 [file 44318_2025_383_MOESM5_ESM.zip › Figure 3/3A/T2D/AcT1.tif]

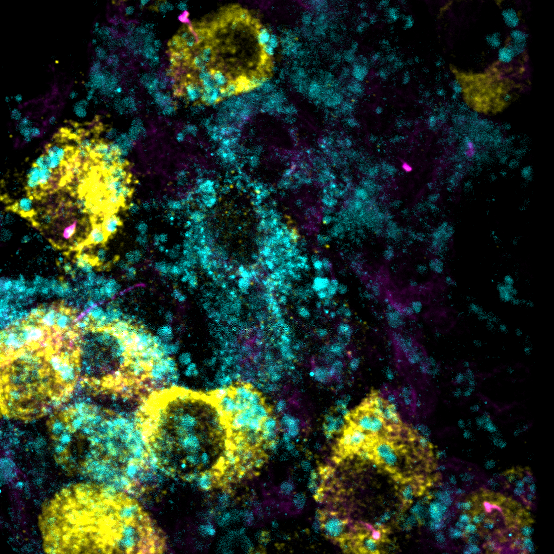

Supplement: Supplementary file 5 — Source data Fig. 3 [file 44318_2025_383_MOESM5_ESM.zip › Figure 3/3A/T2D/MAX_Composite-15.tif (RGB).tif]

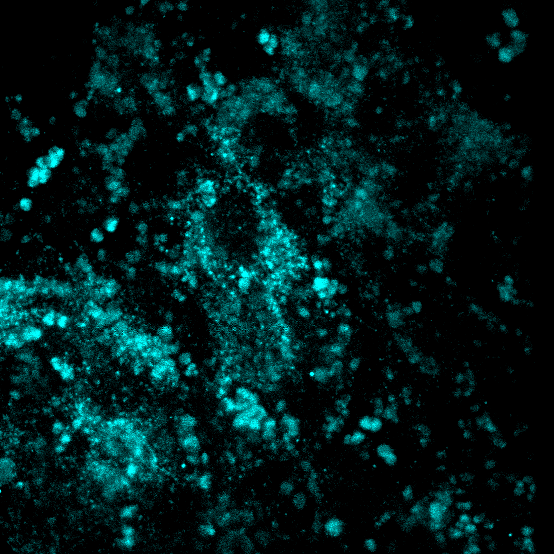

Supplement: Supplementary file 5 — Source data Fig. 3 [file 44318_2025_383_MOESM5_ESM.zip › Figure 3/3A/T2D/Ins1.tif]

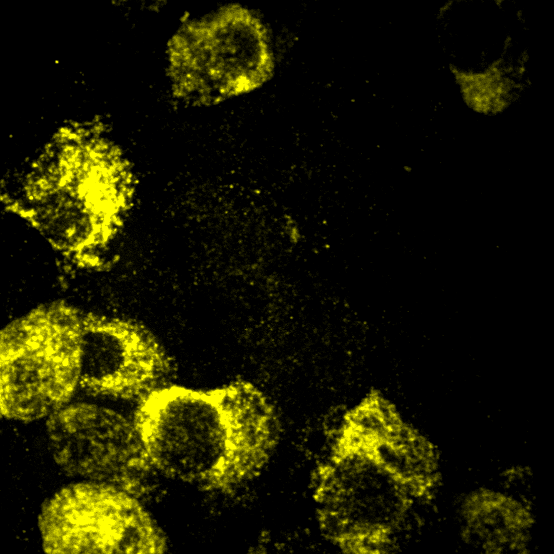

Supplement: Supplementary file 5 — Source data Fig. 3 [file 44318_2025_383_MOESM5_ESM.zip › Figure 3/3A/T2D/sst1.tif]

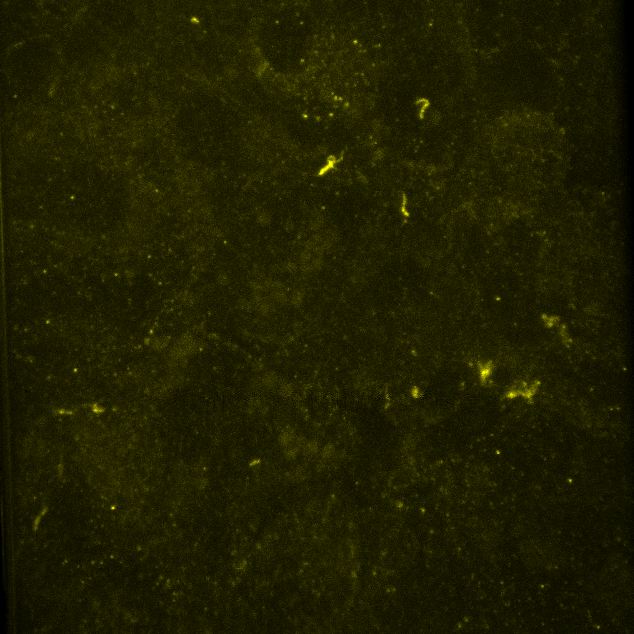

Supplement: Supplementary file 5 — Source data Fig. 3 [file 44318_2025_383_MOESM5_ESM.zip › Figure 3/3N/Non-diabetic/sstr3-3.tif]

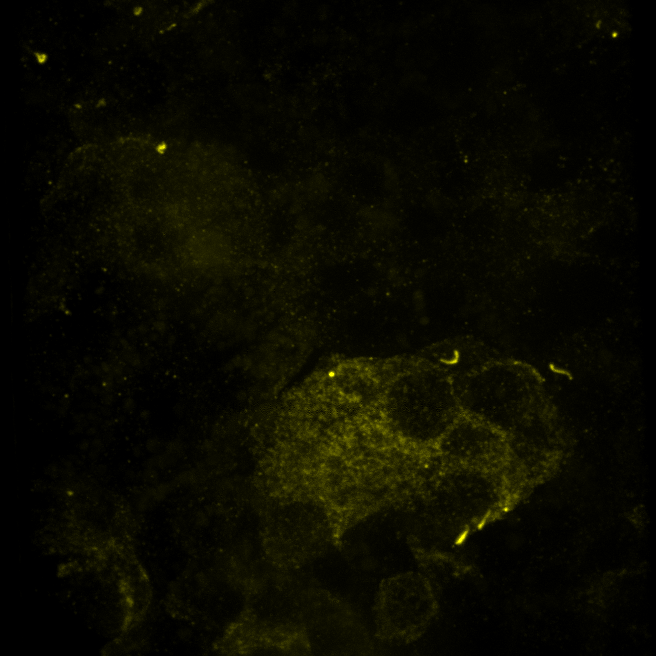

Supplement: Supplementary file 5 — Source data Fig. 3 [file 44318_2025_383_MOESM5_ESM.zip › Figure 3/3N/Non-diabetic/sstr5-4.tif]

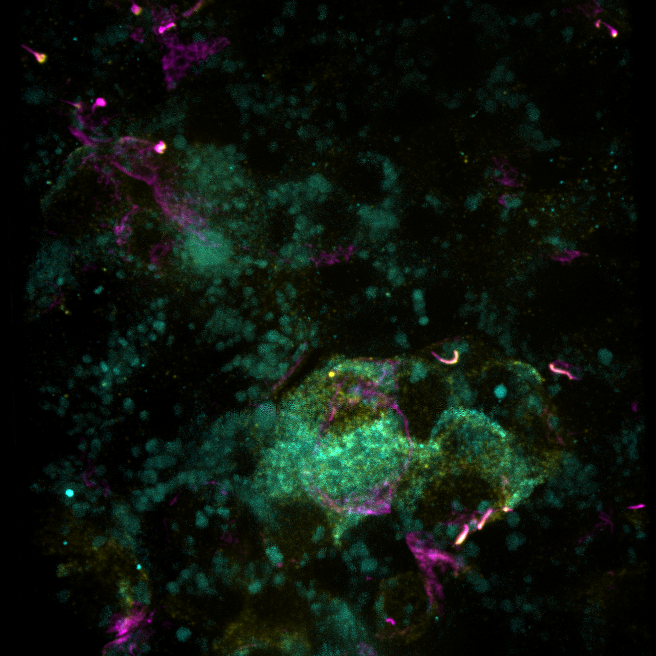

Supplement: Supplementary file 5 — Source data Fig. 3 [file 44318_2025_383_MOESM5_ESM.zip › Figure 3/3N/Non-diabetic/merge4.tif]

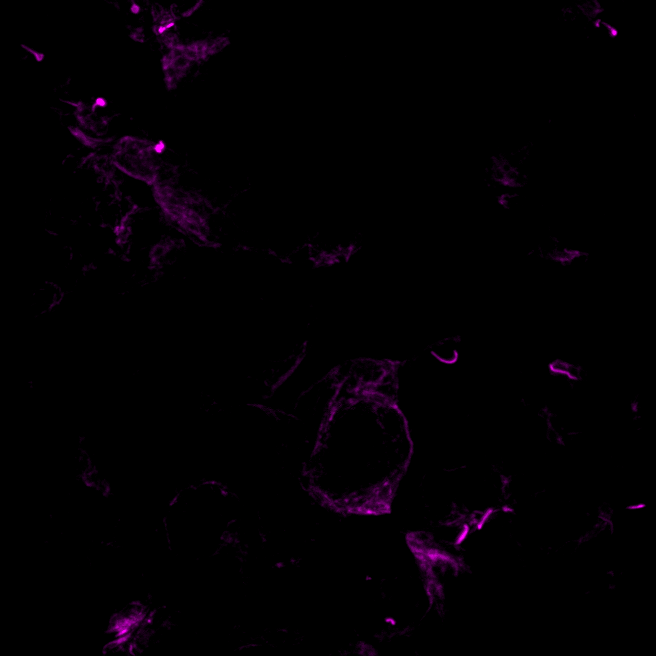

Supplement: Supplementary file 5 — Source data Fig. 3 [file 44318_2025_383_MOESM5_ESM.zip › Figure 3/3N/Non-diabetic/cilia4.tif]

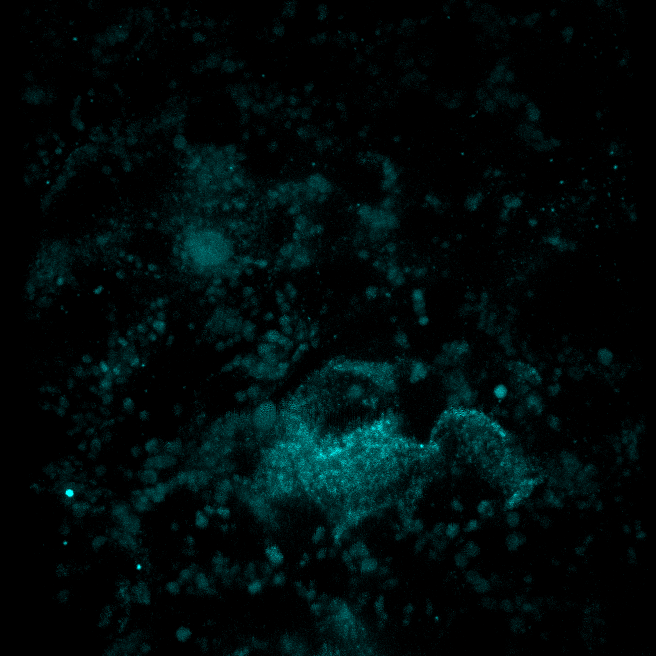

Supplement: Supplementary file 5 — Source data Fig. 3 [file 44318_2025_383_MOESM5_ESM.zip › Figure 3/3N/Non-diabetic/ins4.tif]

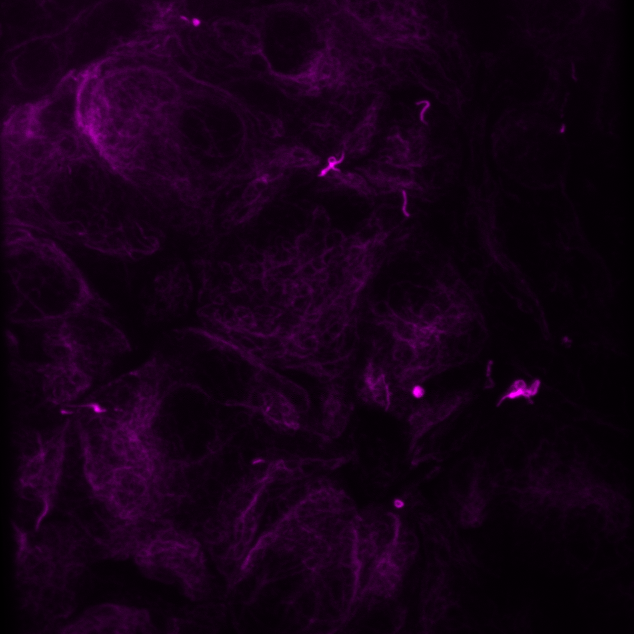

Supplement: Supplementary file 5 — Source data Fig. 3 [file 44318_2025_383_MOESM5_ESM.zip › Figure 3/3N/Non-diabetic/cilia3.tif]

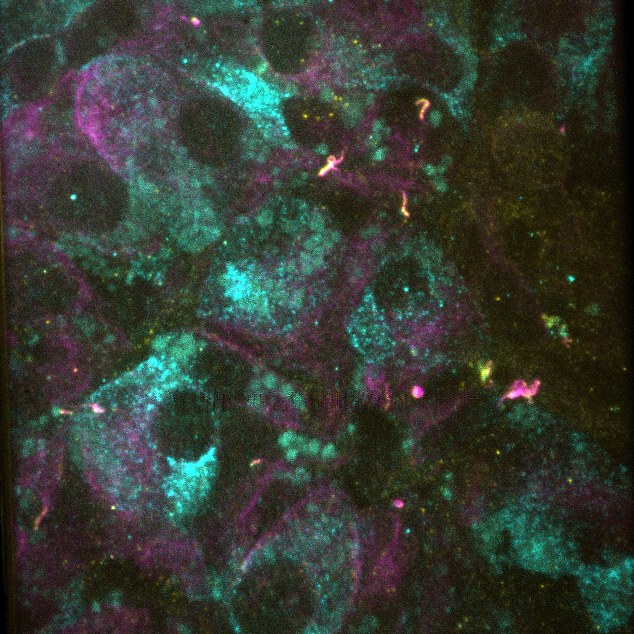

Supplement: Supplementary file 5 — Source data Fig. 3 [file 44318_2025_383_MOESM5_ESM.zip › Figure 3/3N/Non-diabetic/merge3.tif]

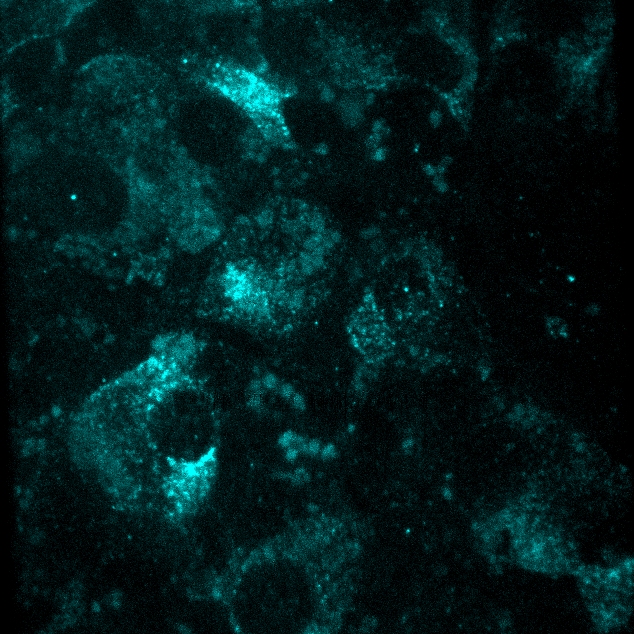

Supplement: Supplementary file 5 — Source data Fig. 3 [file 44318_2025_383_MOESM5_ESM.zip › Figure 3/3N/Non-diabetic/ins3.tif]

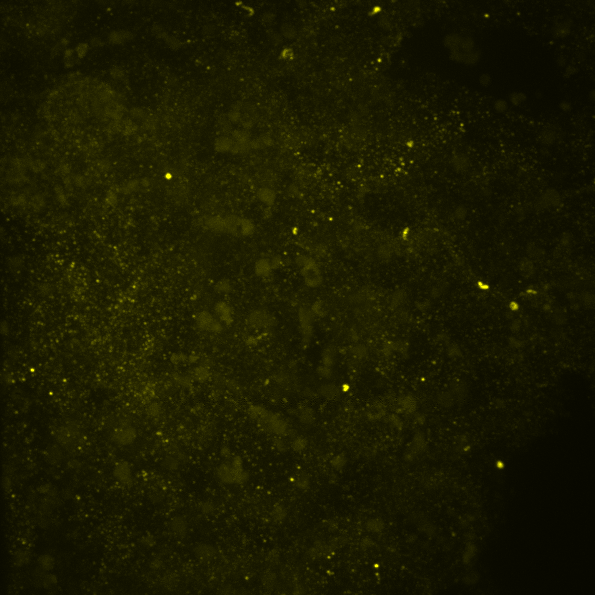

Supplement: Supplementary file 5 — Source data Fig. 3 [file 44318_2025_383_MOESM5_ESM.zip › Figure 3/3N/T2D/sstr3-3.tif]

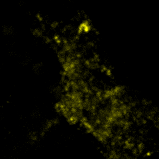

Supplement: Supplementary file 5 — Source data Fig. 3 [file 44318_2025_383_MOESM5_ESM.zip › Figure 3/3N/T2D/sstr5-4.tif]

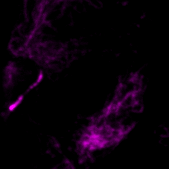

Supplement: Supplementary file 5 — Source data Fig. 3 [file 44318_2025_383_MOESM5_ESM.zip › Figure 3/3N/T2D/cilia5.tif]

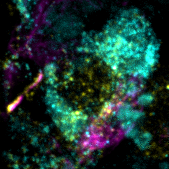

Supplement: Supplementary file 5 — Source data Fig. 3 [file 44318_2025_383_MOESM5_ESM.zip › Figure 3/3N/T2D/merge5.tif]

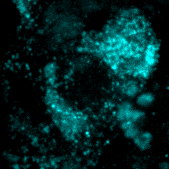

Supplement: Supplementary file 5 — Source data Fig. 3 [file 44318_2025_383_MOESM5_ESM.zip › Figure 3/3N/T2D/ins5.tif]

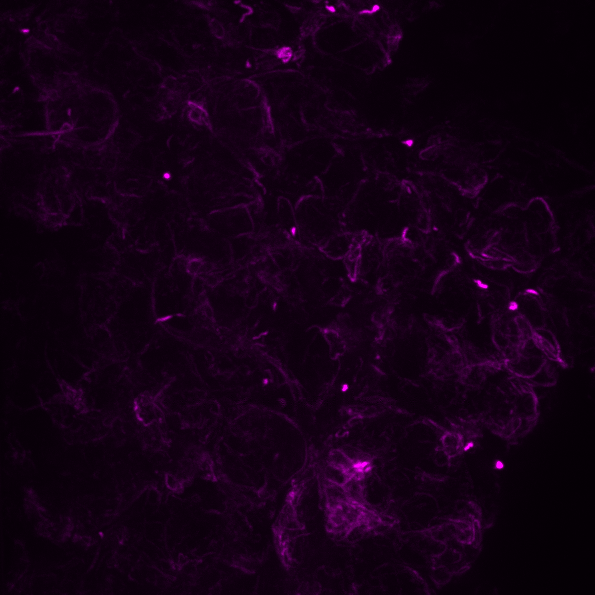

Supplement: Supplementary file 5 — Source data Fig. 3 [file 44318_2025_383_MOESM5_ESM.zip › Figure 3/3N/T2D/cilia3.tif]

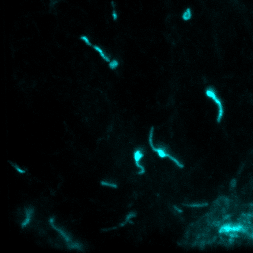

Supplement: Supplementary file 6 — Source data Fig. 4 [file 44318_2025_383_MOESM6_ESM.zip › Figure 4/4E/acetylated tubulin.tif]

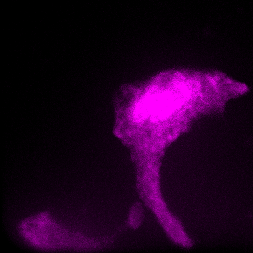

Supplement: Supplementary file 6 — Source data Fig. 4 [file 44318_2025_383_MOESM6_ESM.zip › Figure 4/4E/somatostatin.tif]

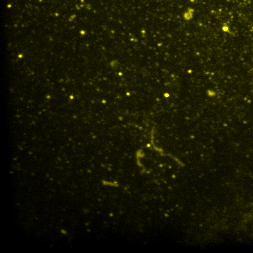

Supplement: Supplementary file 6 — Source data Fig. 4 [file 44318_2025_383_MOESM6_ESM.zip › Figure 4/4E/SSTR3.tif]

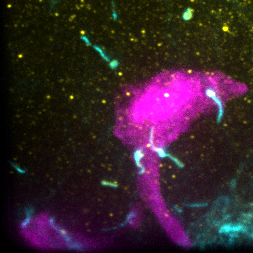

Supplement: Supplementary file 6 — Source data Fig. 4 [file 44318_2025_383_MOESM6_ESM.zip › Figure 4/4E/Composite-2 (RGB).tif]

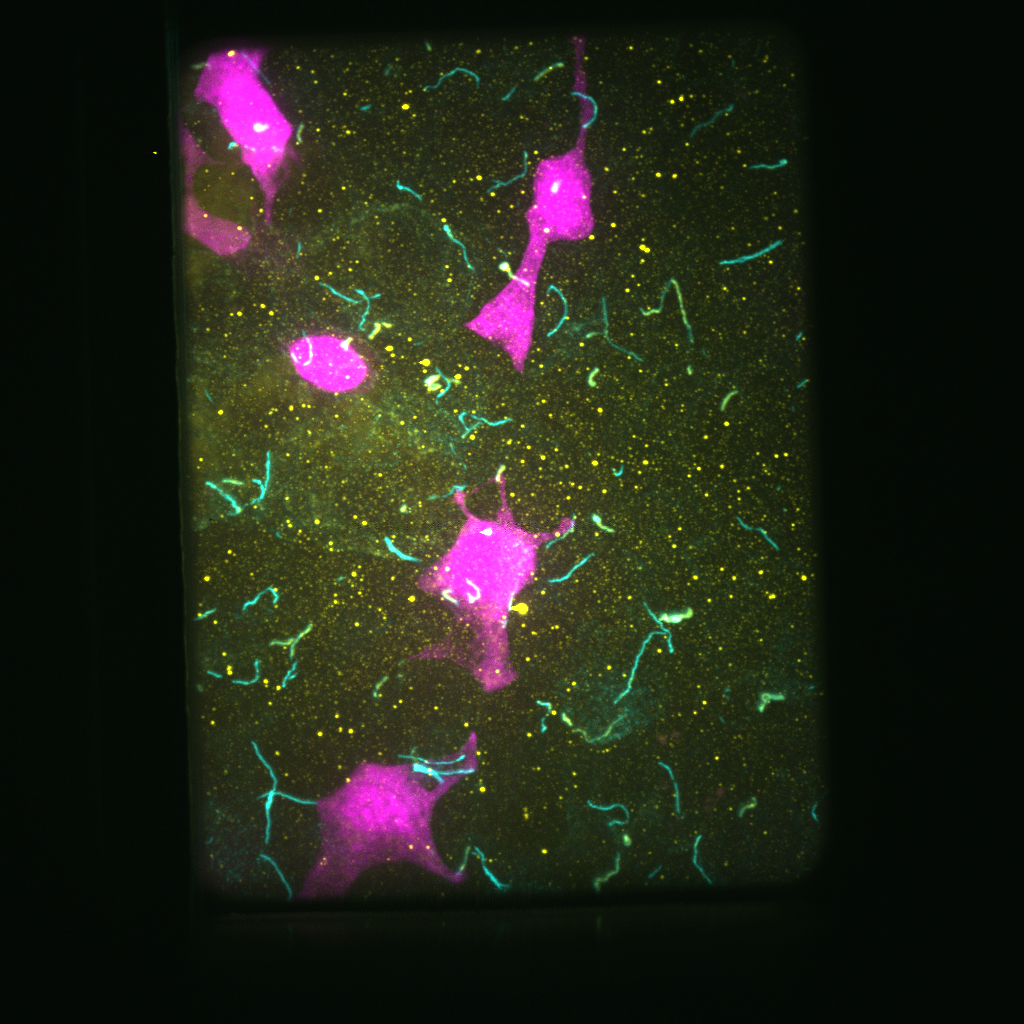

Supplement: Supplementary file 6 — Source data Fig. 4 [file 44318_2025_383_MOESM6_ESM.zip › Figure 4/4C/Composite-1 (RGB).tif]

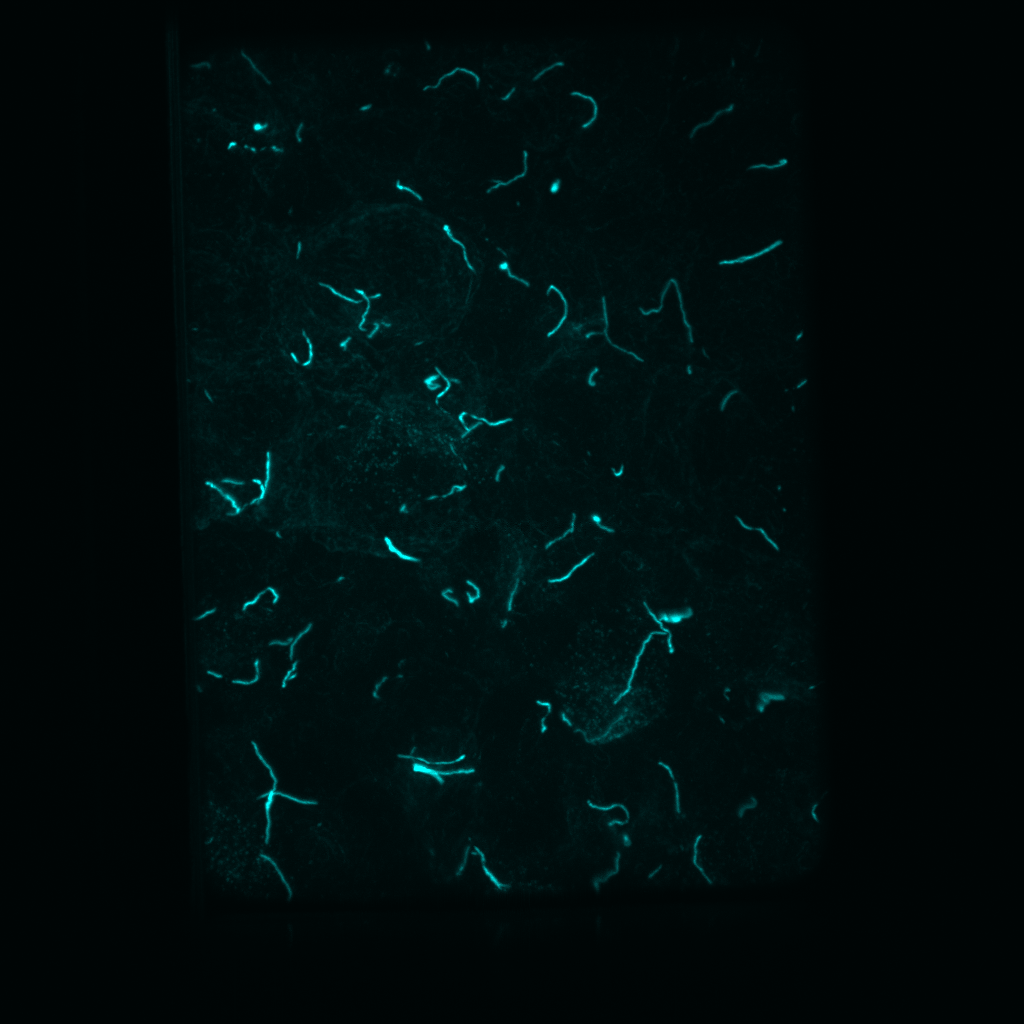

Supplement: Supplementary file 6 — Source data Fig. 4 [file 44318_2025_383_MOESM6_ESM.zip › Figure 4/4C/Acetylated tubulin.tif]

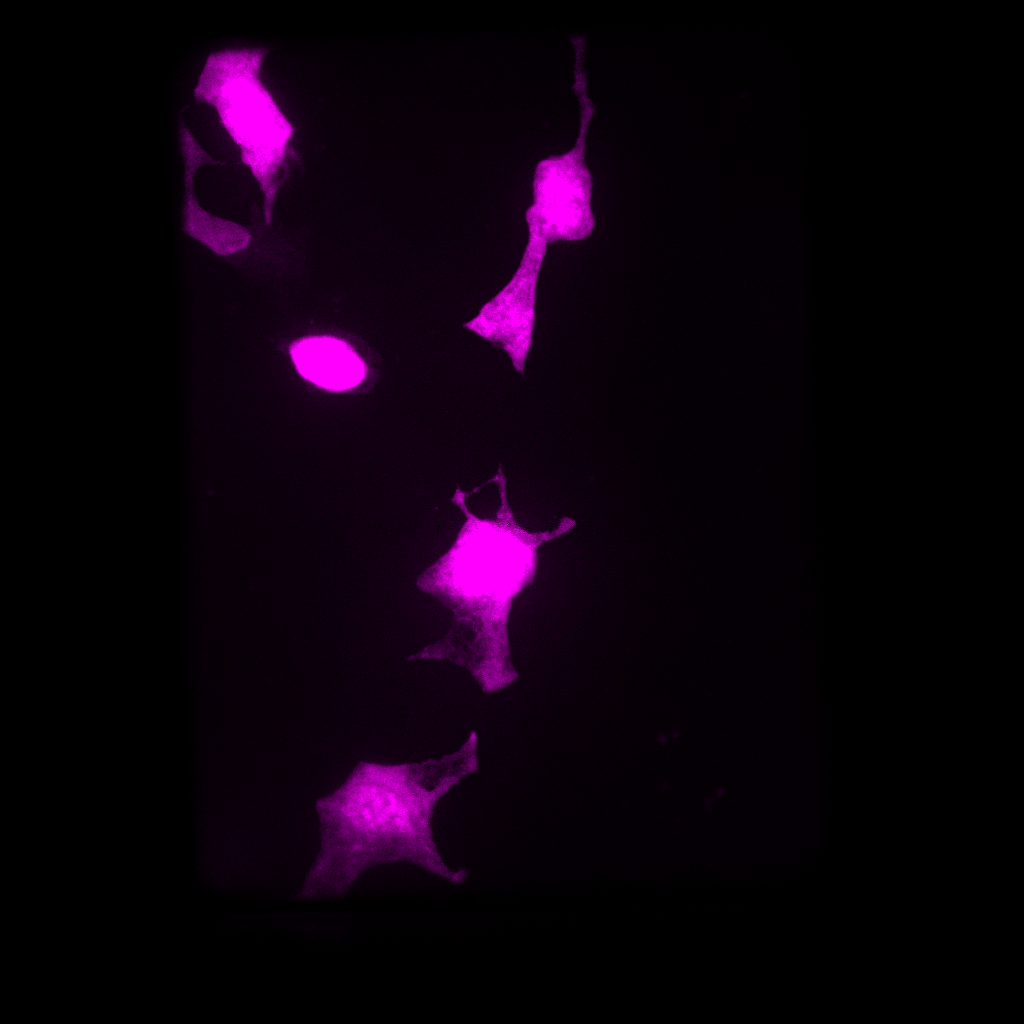

Supplement: Supplementary file 6 — Source data Fig. 4 [file 44318_2025_383_MOESM6_ESM.zip › Figure 4/4C/Somatostatin.tif]

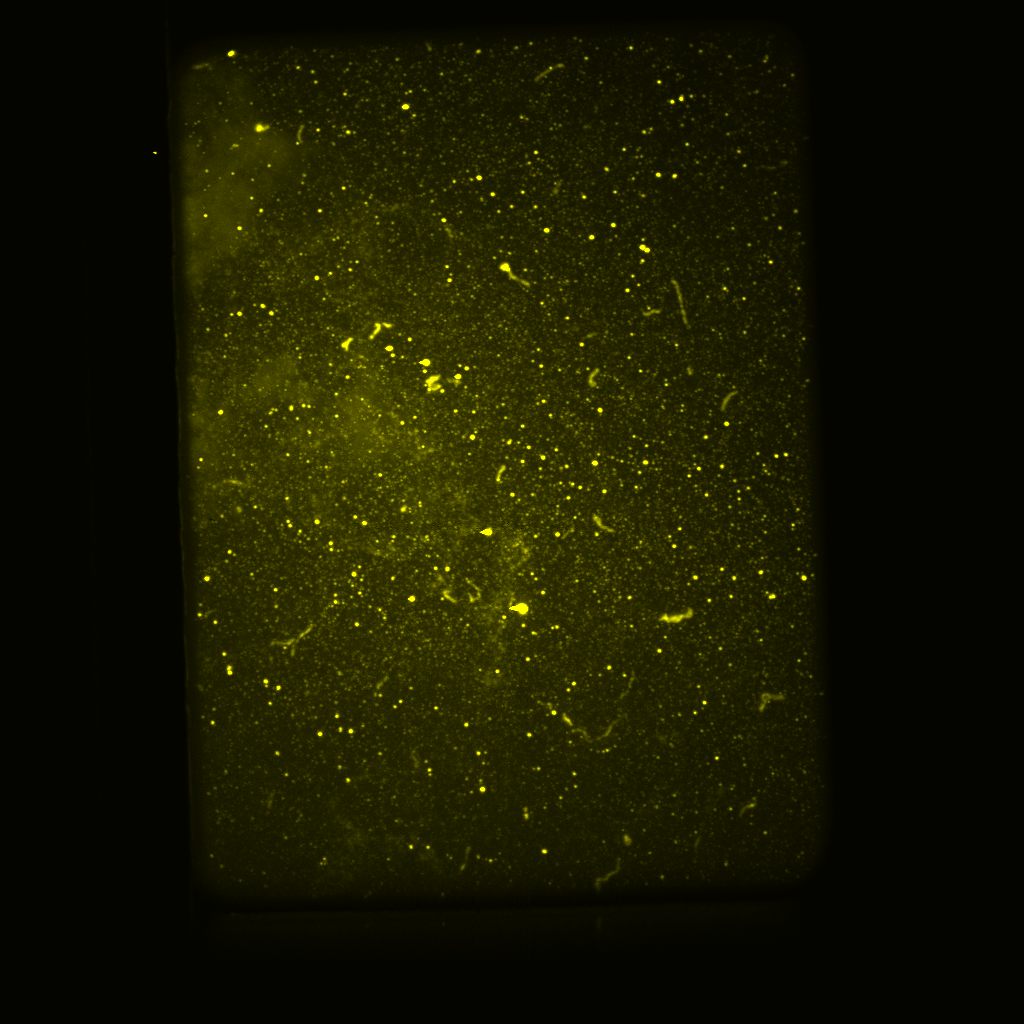

Supplement: Supplementary file 6 — Source data Fig. 4 [file 44318_2025_383_MOESM6_ESM.zip › Figure 4/4C/SSTR3.tif]

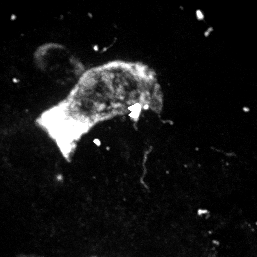

Supplement: Supplementary file 6 — Source data Fig. 4 [file 44318_2025_383_MOESM6_ESM.zip › Figure 4/4A/somatostatin.tif]

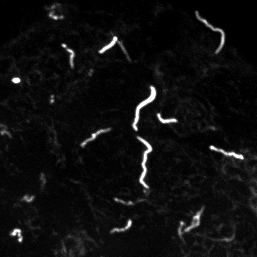

Supplement: Supplementary file 6 — Source data Fig. 4 [file 44318_2025_383_MOESM6_ESM.zip › Figure 4/4A/acetylated-tubulin.tif]

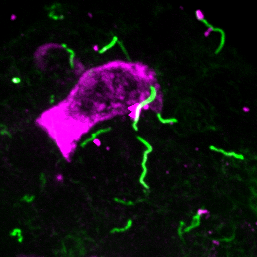

Supplement: Supplementary file 6 — Source data Fig. 4 [file 44318_2025_383_MOESM6_ESM.zip › Figure 4/4A/merge1.tif]

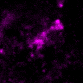

Supplement: Supplementary file 6 — Source data Fig. 4 [file 44318_2025_383_MOESM6_ESM.zip › Figure 4/4F/C2-MAX_Composite-1.tif]

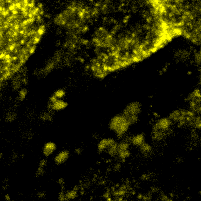

Supplement: Supplementary file 6 — Source data Fig. 4 [file 44318_2025_383_MOESM6_ESM.zip › Figure 4/4F/C1-MAX_Composite-2.tif]

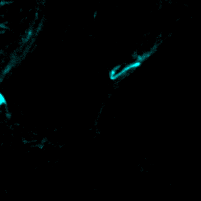

Supplement: Supplementary file 6 — Source data Fig. 4 [file 44318_2025_383_MOESM6_ESM.zip › Figure 4/4F/C2-MAX_Composite-2.tif]

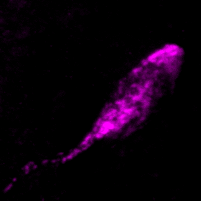

Supplement: Supplementary file 6 — Source data Fig. 4 [file 44318_2025_383_MOESM6_ESM.zip › Figure 4/4F/C3-MAX_Composite-2.tif]

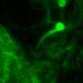

Supplement: Supplementary file 6 — Source data Fig. 4 [file 44318_2025_383_MOESM6_ESM.zip › Figure 4/4F/C1-MAX_Composite-1.tif]

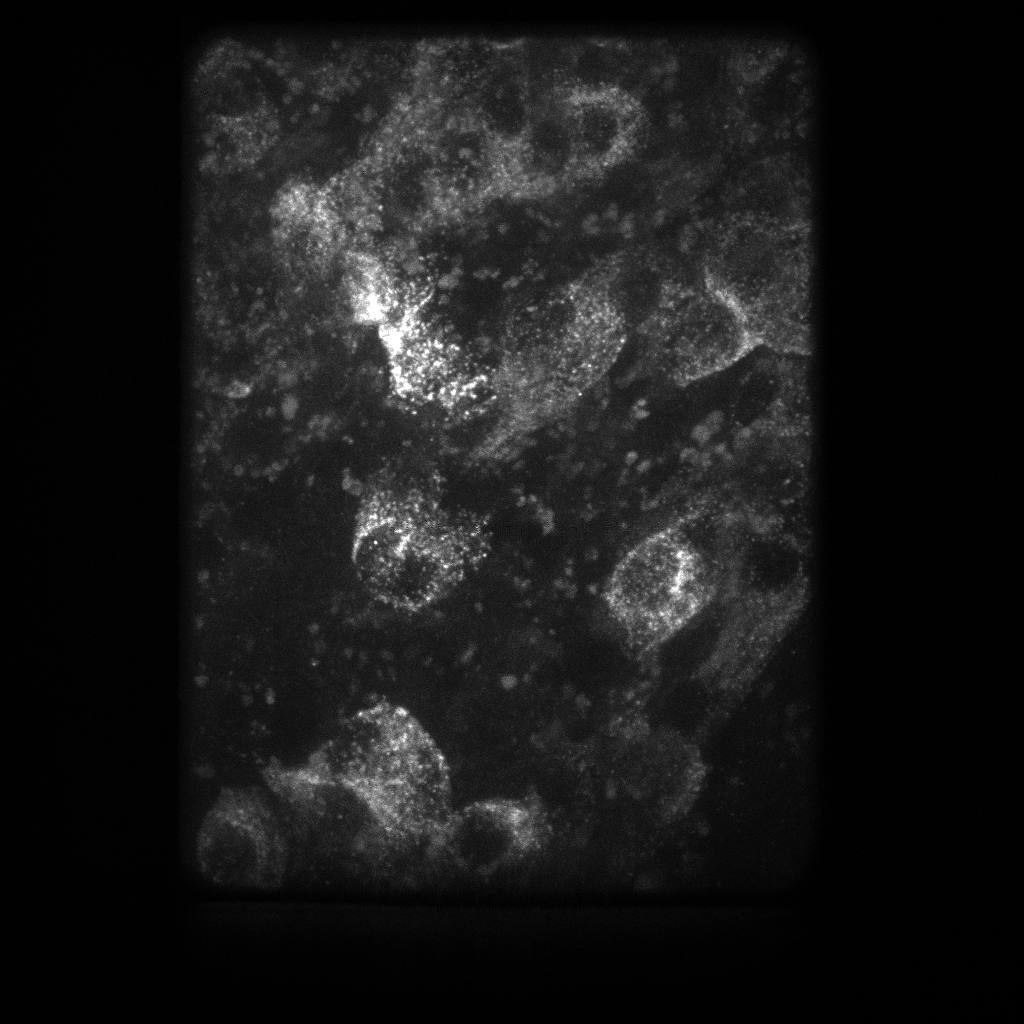

Supplement: Supplementary file 6 — Source data Fig. 4 [file 44318_2025_383_MOESM6_ESM.zip › Figure 4/4F/ND-islet_SST-INS-AcTub.tif]

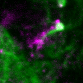

Supplement: Supplementary file 6 — Source data Fig. 4 [file 44318_2025_383_MOESM6_ESM.zip › Figure 4/4F/MAX_Composite-1 (RGB).tif]

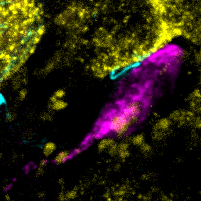

Supplement: Supplementary file 6 — Source data Fig. 4 [file 44318_2025_383_MOESM6_ESM.zip › Figure 4/4F/Composite (RGB).tif]

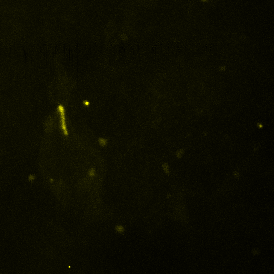

Supplement: Supplementary file 6 — Source data Fig. 4 [file 44318_2025_383_MOESM6_ESM.zip › Figure 4/4H/sst1-sensor-delta.tif]

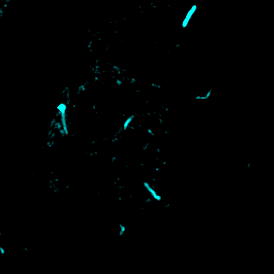

Supplement: Supplementary file 6 — Source data Fig. 4 [file 44318_2025_383_MOESM6_ESM.zip › Figure 4/4H/acetylated tubulin.tif]

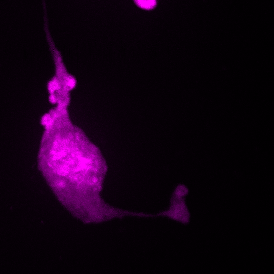

Supplement: Supplementary file 6 — Source data Fig. 4 [file 44318_2025_383_MOESM6_ESM.zip › Figure 4/4H/tdtomato-SST.tif]

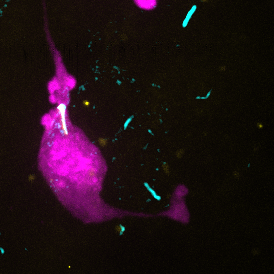

Supplement: Supplementary file 6 — Source data Fig. 4 [file 44318_2025_383_MOESM6_ESM.zip › Figure 4/4H/Composite (RGB).tif]

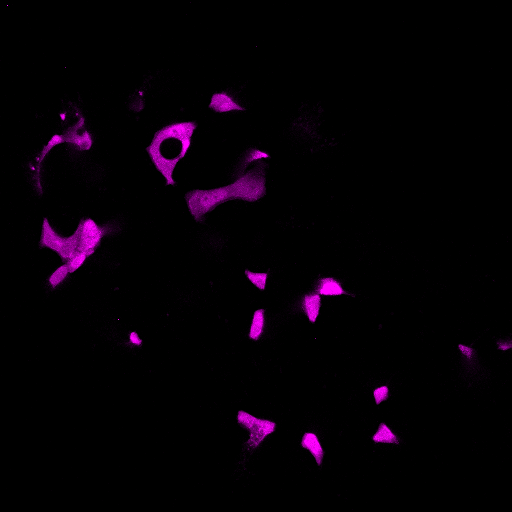

Supplement: Supplementary file 6 — Source data Fig. 4 [file 44318_2025_383_MOESM6_ESM.zip › Figure 4/4I/tdtomato-SST.tif]

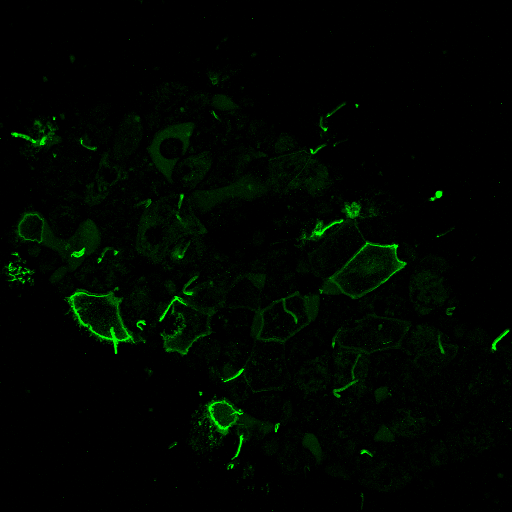

Supplement: Supplementary file 6 — Source data Fig. 4 [file 44318_2025_383_MOESM6_ESM.zip › Figure 4/4I/SST1-sensor.tif]

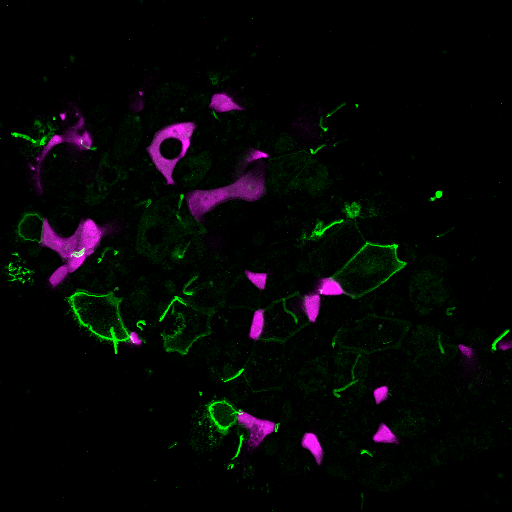

Supplement: Supplementary file 6 — Source data Fig. 4 [file 44318_2025_383_MOESM6_ESM.zip › Figure 4/4I/Merged-1 (RGB).tif]

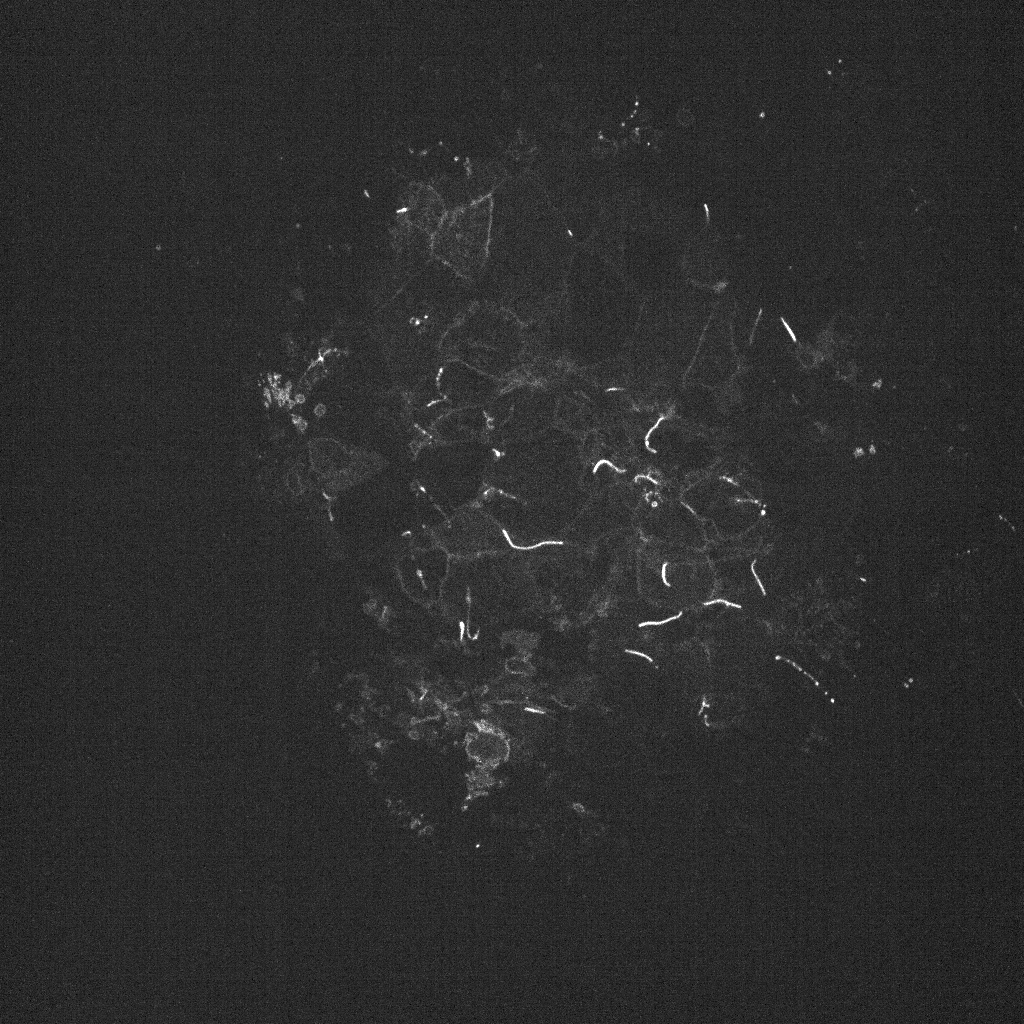

Supplement: Supplementary file 6 — Source data Fig. 4 [file 44318_2025_383_MOESM6_ESM.zip › Figure 4/4G/mouse islet SST1.tif]

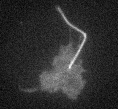

Supplement: Supplementary file 7 — Source data Fig. 5 [file 44318_2025_383_MOESM7_ESM.zip › Figure 5/5A/2017-09-06 no 2b ACCEPTOR.tif]

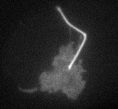

Supplement: Supplementary file 7 — Source data Fig. 5 [file 44318_2025_383_MOESM7_ESM.zip › Figure 5/5A/2017-09-06 no 2b DONOR.tif]

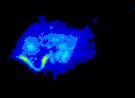

Supplement: Supplementary file 7 — Source data Fig. 5 [file 44318_2025_383_MOESM7_ESM.zip › Figure 5/5C/low cAMP.jpg]

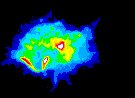

Supplement: Supplementary file 7 — Source data Fig. 5 [file 44318_2025_383_MOESM7_ESM.zip › Figure 5/5C/high cAMP.jpg]

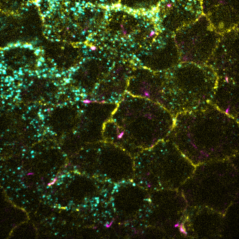

Supplement: Supplementary file 7 — Source data Fig. 5 [file 44318_2025_383_MOESM7_ESM.zip › Figure 5/5G/GLP-1 15 min/Composite (RGB)-1.tif]

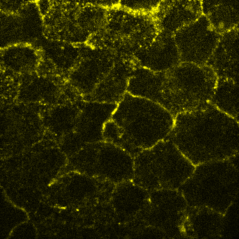

Supplement: Supplementary file 7 — Source data Fig. 5 [file 44318_2025_383_MOESM7_ESM.zip › Figure 5/5G/GLP-1 15 min/GLP1R-1.tif]

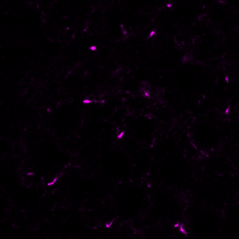

Supplement: Supplementary file 7 — Source data Fig. 5 [file 44318_2025_383_MOESM7_ESM.zip › Figure 5/5G/GLP-1 15 min/GABBR1-1.tif]

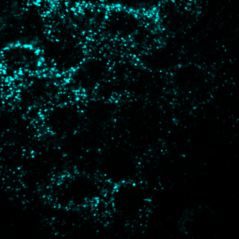

Supplement: Supplementary file 7 — Source data Fig. 5 [file 44318_2025_383_MOESM7_ESM.zip › Figure 5/5G/GLP-1 15 min/insulin-1.tif]

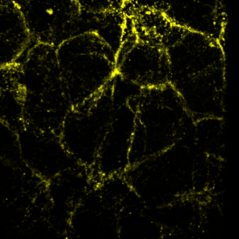

Supplement: Supplementary file 7 — Source data Fig. 5 [file 44318_2025_383_MOESM7_ESM.zip › Figure 5/5G/Control/MAX_glp1r-1.tif]

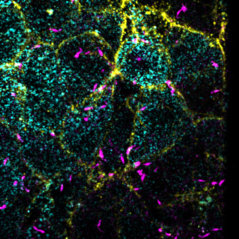

Supplement: Supplementary file 7 — Source data Fig. 5 [file 44318_2025_383_MOESM7_ESM.zip › Figure 5/5G/Control/MAX-Composite (RGB)-1.tif]

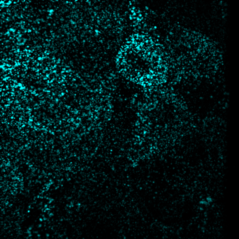

Supplement: Supplementary file 7 — Source data Fig. 5 [file 44318_2025_383_MOESM7_ESM.zip › Figure 5/5G/Control/MAX_insulin-1.tif]

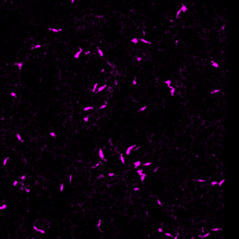

Supplement: Supplementary file 7 — Source data Fig. 5 [file 44318_2025_383_MOESM7_ESM.zip › Figure 5/5G/Control/MAX_cilia-1.tif]

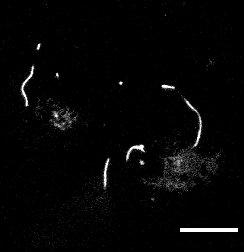

Supplement: Supplementary file 9 — Source data Fig. 7 [file 44318_2025_383_MOESM9_ESM.zip › Figure 7/7B/min6PI 5HT6-GGECO.jpg]

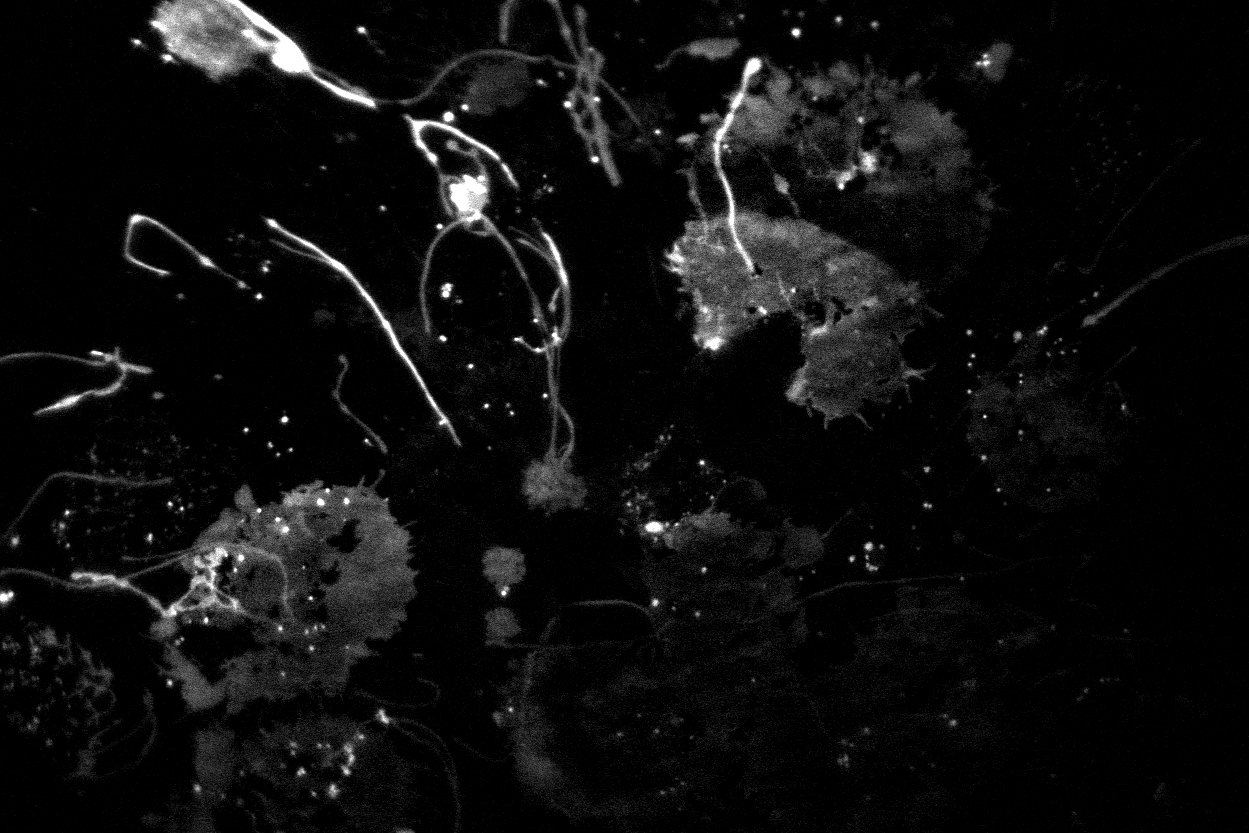

Supplement: Supplementary file 9 — Source data Fig. 7 [file 44318_2025_383_MOESM9_ESM.zip › Figure 7/7K/islet-5HT6-GGECO.tif]

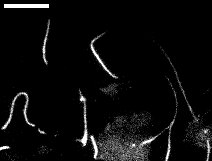

Supplement: Supplementary file 9 — Source data Fig. 7 [file 44318_2025_383_MOESM9_ESM.zip › Figure 7/7A/mouse islet (new obj) 5HT6-GGECO.jpg]

## Slide 1
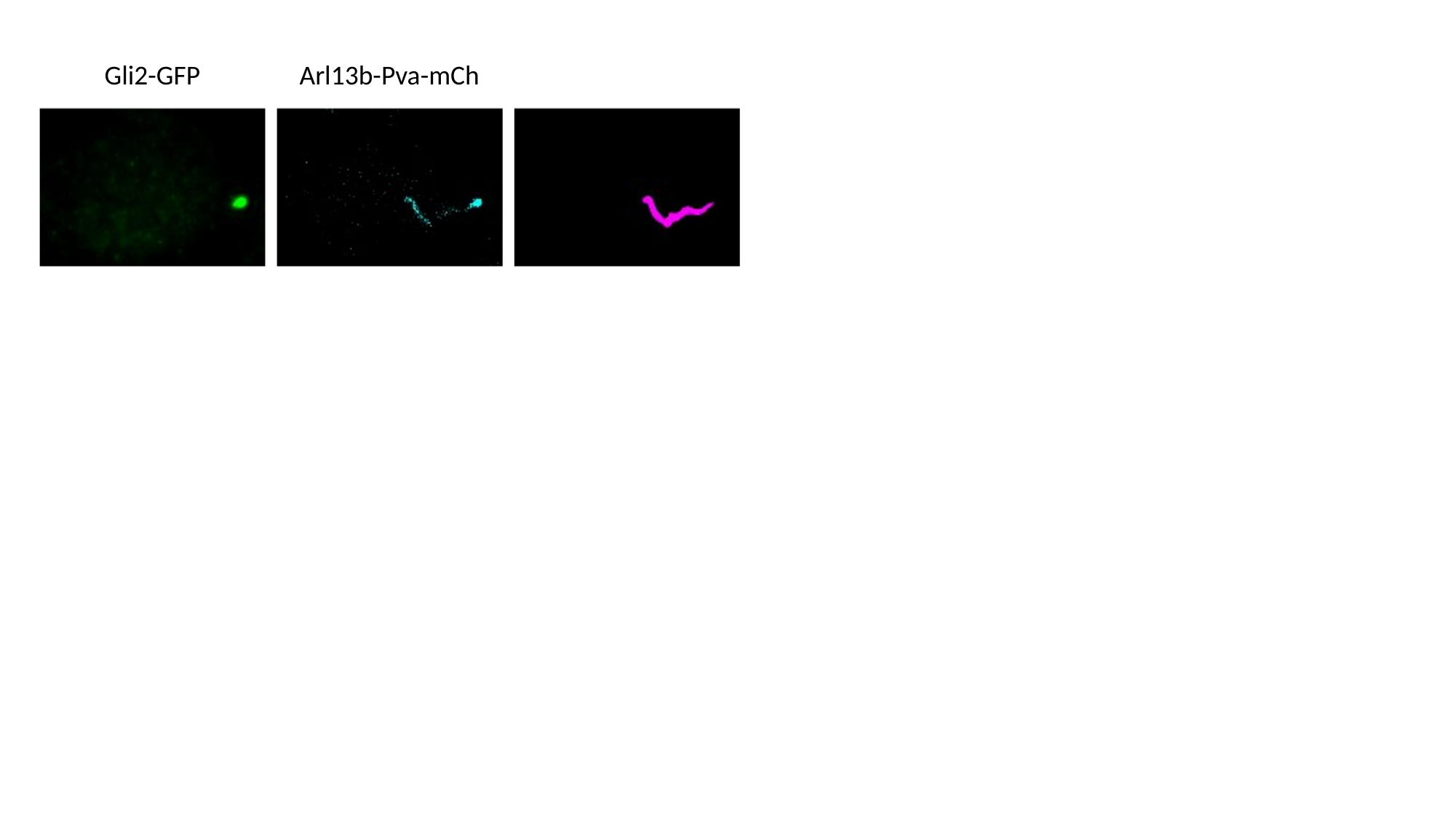

Gli2-GFP
Arl13b-Pva-mCh

Supplement: Supplementary file 10 — Source data Fig. 8 [file 44318_2025_383_MOESM10_ESM.zip › Figure 8/8I/sponge images.pptx]

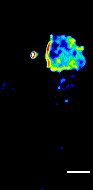

Supplement: Supplementary file 10 — Source data Fig. 8 [file 44318_2025_383_MOESM10_ESM.zip › Figure 8/8M/basal.jpg]

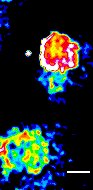

Supplement: Supplementary file 10 — Source data Fig. 8 [file 44318_2025_383_MOESM10_ESM.zip › Figure 8/8M/GLP-1.jpg]

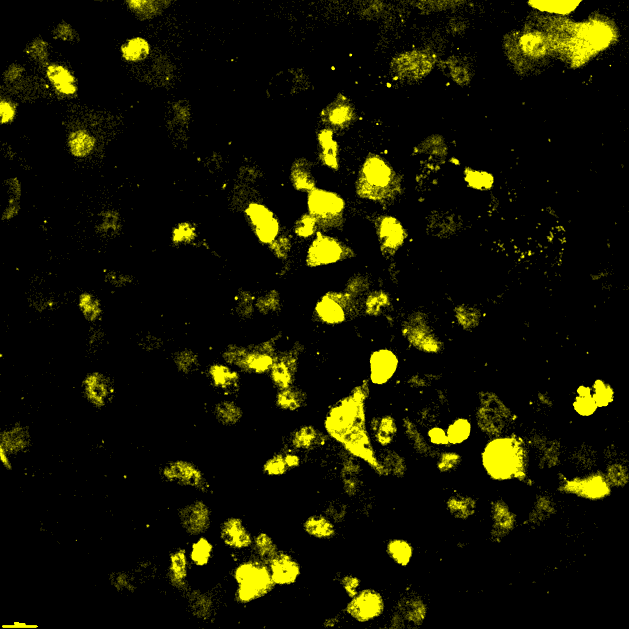

Supplement: Supplementary file 10 — Source data Fig. 8 [file 44318_2025_383_MOESM10_ESM.zip › Figure 8/8A and B/SST/mouse islets Halo-Gli2- SST_Series005_ch03_SV-1.tif]

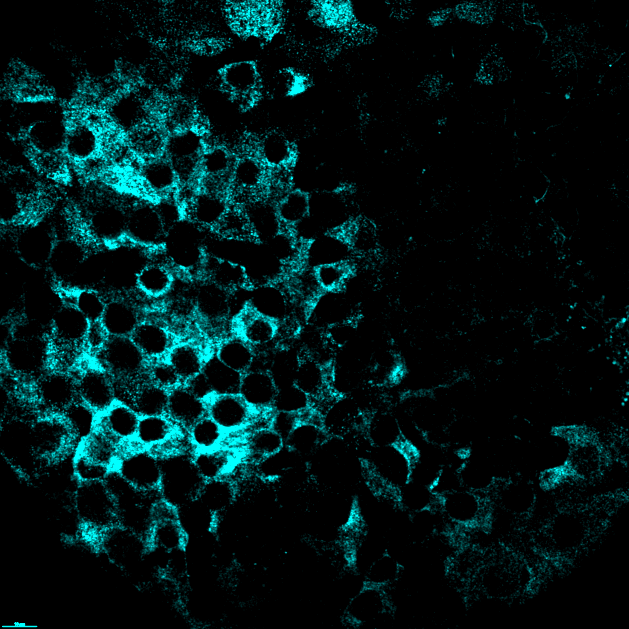

Supplement: Supplementary file 10 — Source data Fig. 8 [file 44318_2025_383_MOESM10_ESM.zip › Figure 8/8A and B/SST/mouse islets Halo-Gli2- SST_Series005_ch02_SV-1.tif]

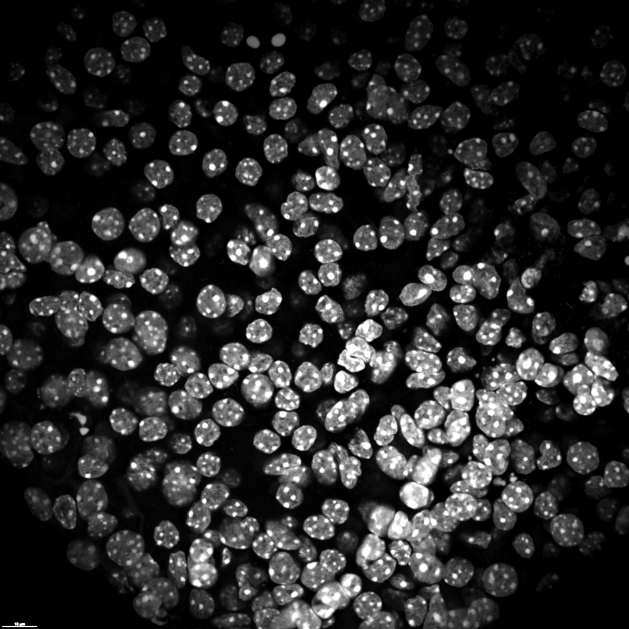

Supplement: Supplementary file 10 — Source data Fig. 8 [file 44318_2025_383_MOESM10_ESM.zip › Figure 8/8A and B/SST/mouse islets Halo-Gli2- SST_Series005_ch00_SV-1.tif]

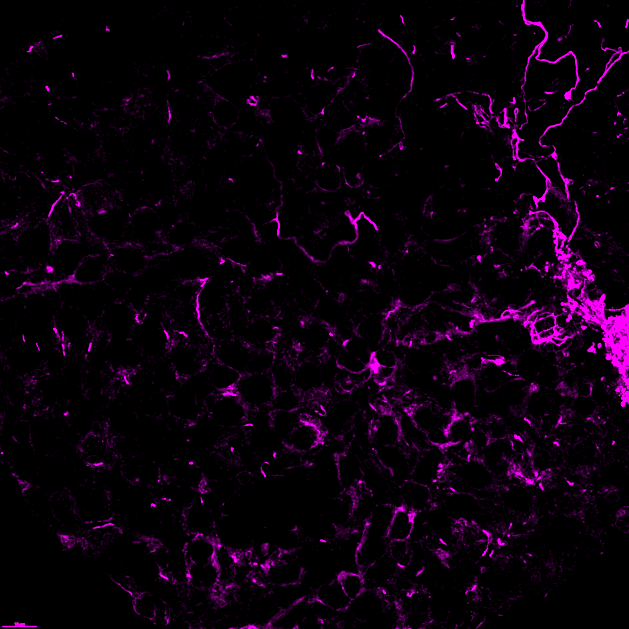

Supplement: Supplementary file 10 — Source data Fig. 8 [file 44318_2025_383_MOESM10_ESM.zip › Figure 8/8A and B/SST/mouse islets Halo-Gli2- SST_Series005_ch01_SV-1.tif]

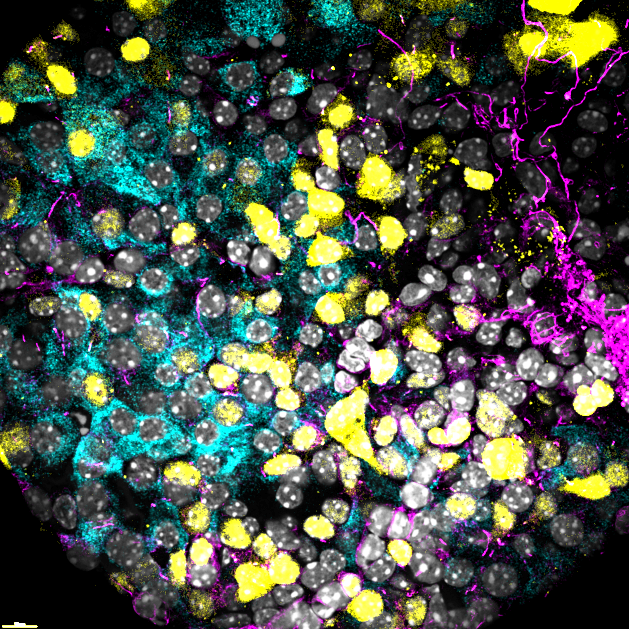

Supplement: Supplementary file 10 — Source data Fig. 8 [file 44318_2025_383_MOESM10_ESM.zip › Figure 8/8A and B/SST/Composite (RGB).tif]

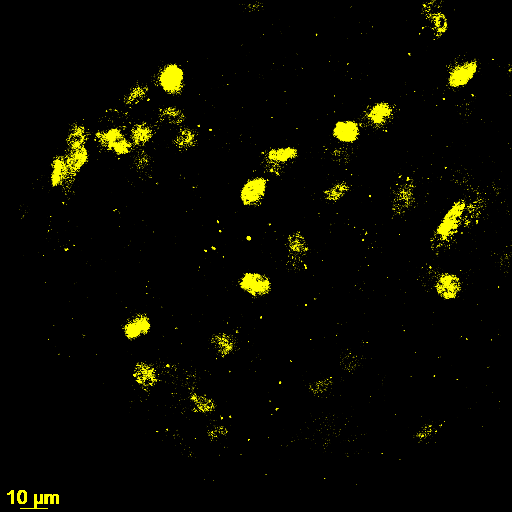

Supplement: Supplementary file 10 — Source data Fig. 8 [file 44318_2025_383_MOESM10_ESM.zip › Figure 8/8A and B/SAG/SAG_Series001_ch03_SV.tif]

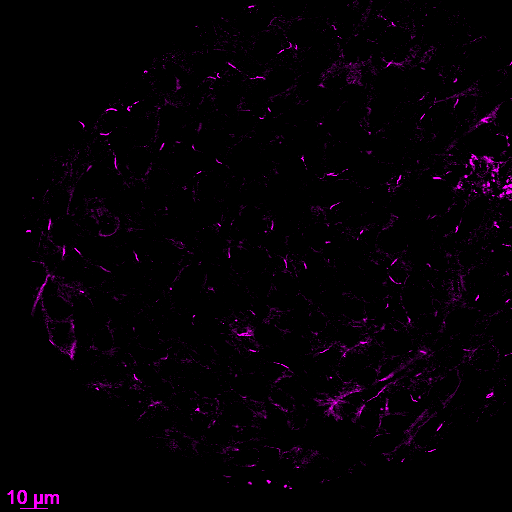

Supplement: Supplementary file 10 — Source data Fig. 8 [file 44318_2025_383_MOESM10_ESM.zip › Figure 8/8A and B/SAG/SAG_Series001_ch01_SV.tif]

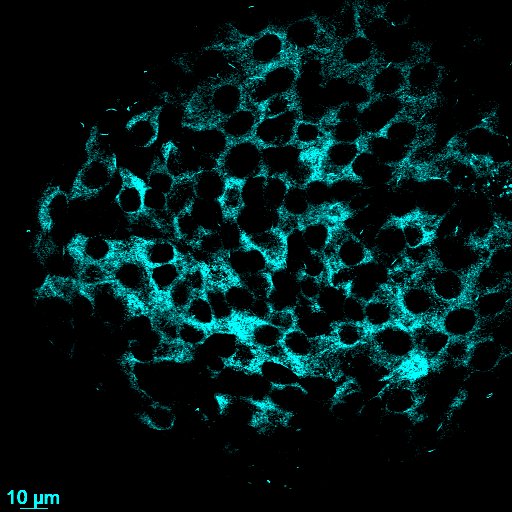

Supplement: Supplementary file 10 — Source data Fig. 8 [file 44318_2025_383_MOESM10_ESM.zip › Figure 8/8A and B/SAG/SAG_Series001_ch02_SV.tif]

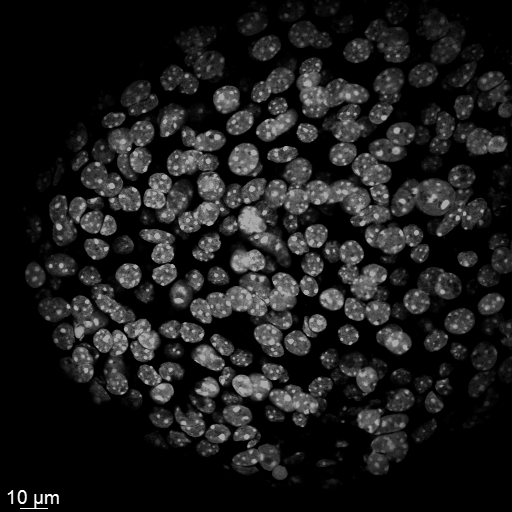

Supplement: Supplementary file 10 — Source data Fig. 8 [file 44318_2025_383_MOESM10_ESM.zip › Figure 8/8A and B/SAG/SAG_Series001_ch00_SV.tif]
