# Supplementary material for: Somatostatin triggers local cAMP and Ca2+ signaling in primary cilia to modulate pancreatic β-cell function
Source: EMBO J. 2025 Feb 12;44(6):1663–91. doi: 10.1038/s44318-025-00383-7 (PMC11914567; doi:10.1038/s44318-025-00383-7)
Supplement: Supplementary file 8 — Source data Fig. 6 [file 44318_2025_383_MOESM8_ESM.zip › Figure 6/6E/SSTR3 KD photos.pptx]

## Slide 1
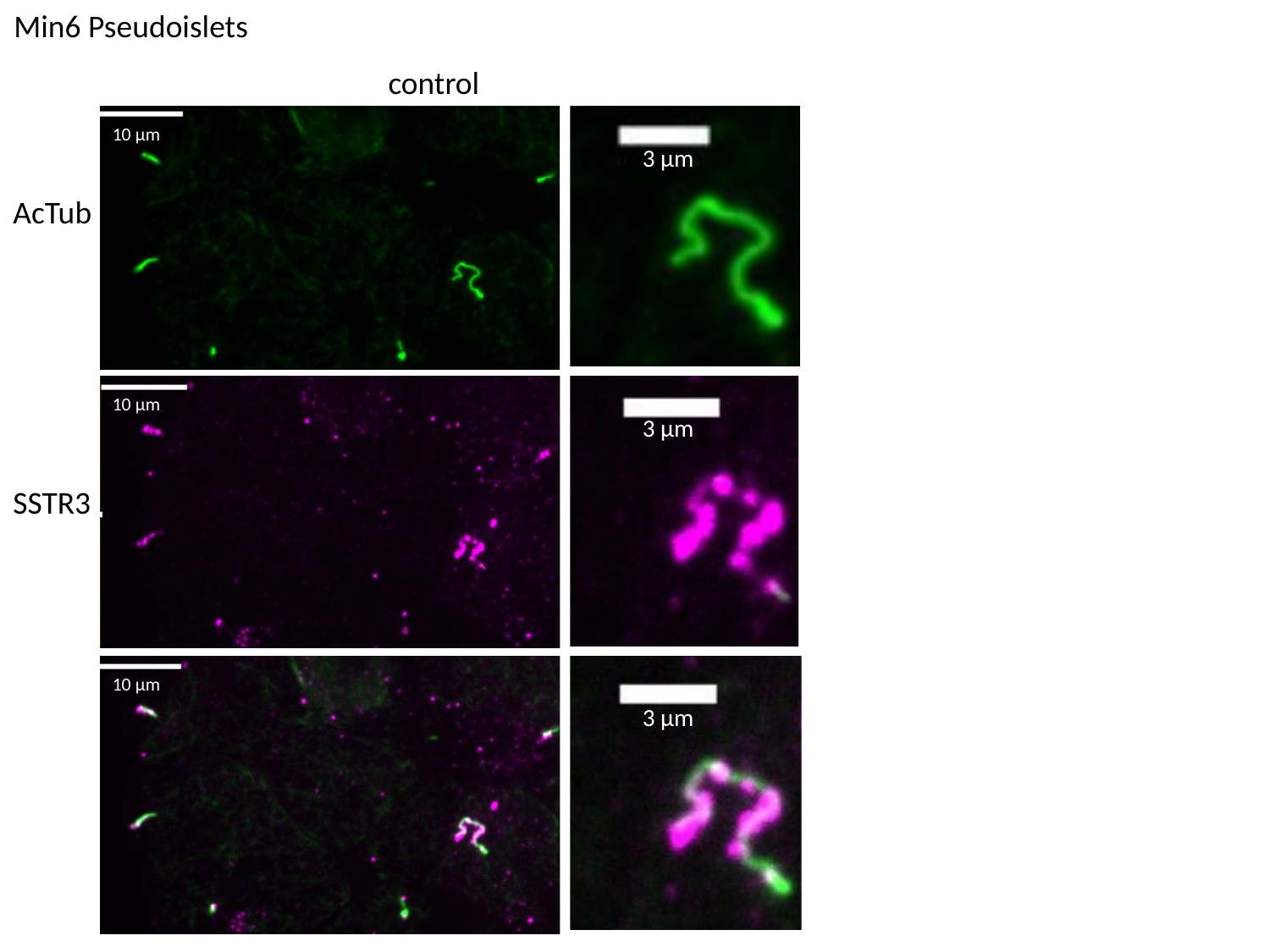

Min6 Pseudoislets
control
10 μm
3 μm
AcTub
10 μm
3 μm
SSTR3
10 μm
3 μm

## Slide 2
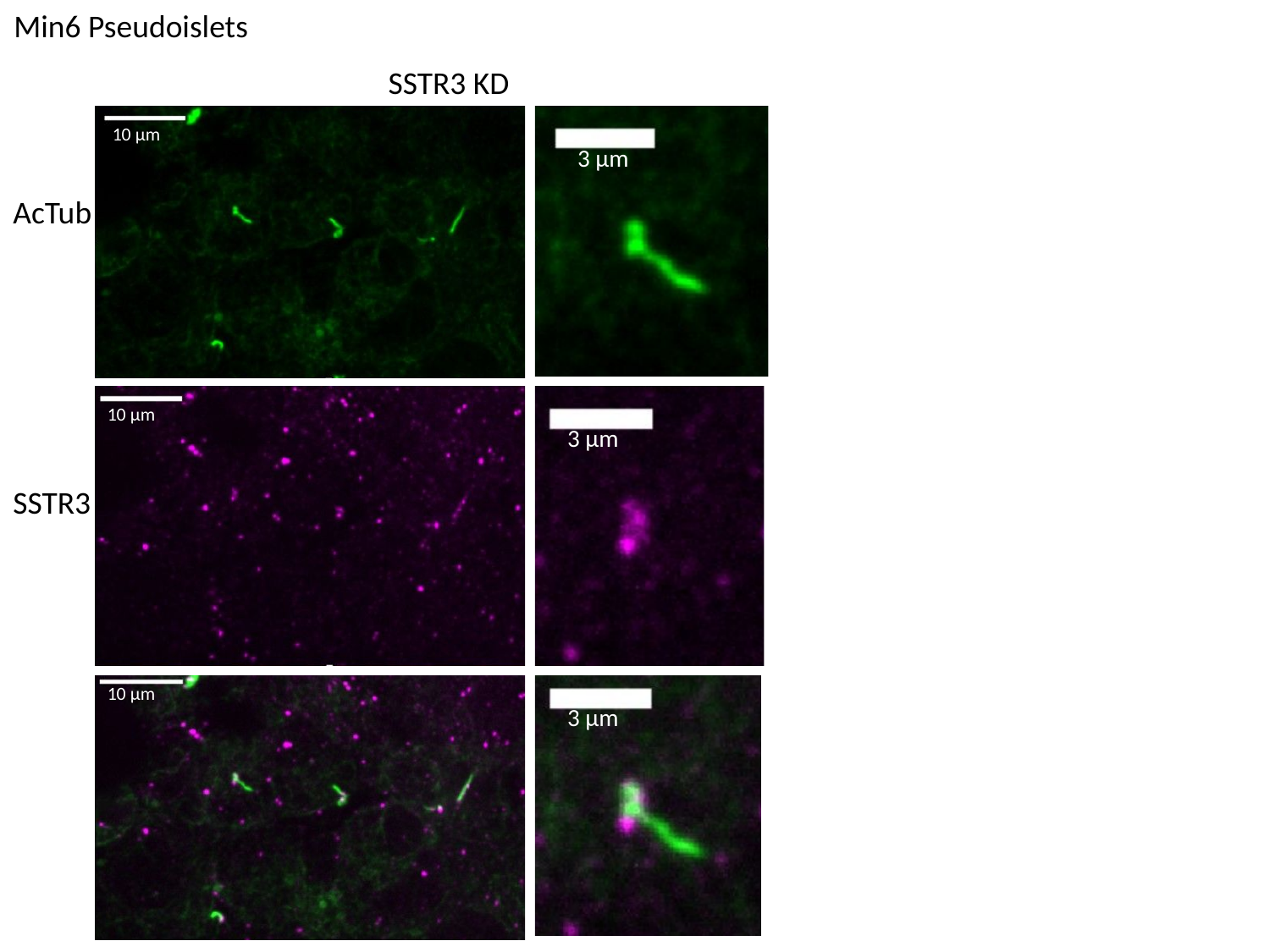

Min6 Pseudoislets
SSTR3 KD
10 μm
3 μm
AcTub
10 μm
3 μm
SSTR3
10 μm
3 μm
