# Supplementary material for: Somatostatin triggers local cAMP and Ca2+ signaling in primary cilia to modulate pancreatic β-cell function
Source: EMBO J. 2025 Feb 12;44(6):1663–91. doi: 10.1038/s44318-025-00383-7 (PMC11914567; doi:10.1038/s44318-025-00383-7)
Supplement: Supplementary file 8 — Source data Fig. 6 [file 44318_2025_383_MOESM8_ESM.zip › Figure 6/6C/TUG stats.docx]

Cilia 37

Cytosol 26 cells

| Šídák's multiple comparisons test | Mean Diff, | 95,00% CI of diff, | Below threshold? | Summary | Adjusted P Value |
| --- | --- | --- | --- | --- | --- |
|  |  |  |  |  |  |
| WT-cilia |  |  |  |  |  |
| basal vs. TUG | -0,01795 | -0,03421 to -0,001685 | Yes | * | 0,0211 |
| basal vs. SST | 0,01141 | -0,01244 to 0,03525 | No | ns | 0,8983 |
| basal vs. SST- | -0,01804 | -0,04921 to 0,01313 | No | ns | 0,7005 |
| basal vs. TUG- | -0,01923 | -0,05741 to 0,01895 | No | ns | 0,8580 |
| basal vs. Fsk 10 | -0,08352 | -0,1409 to -0,02614 | Yes | *** | 0,0009 |
| TUG vs. SST | 0,02935 | 0,01584 to 0,04287 | Yes | **** | <0,0001 |
| TUG vs. SST- | -9,417e-005 | -0,02002 to 0,01984 | No | ns | >0,9999 |
| TUG vs. TUG- | -0,001286 | -0,03053 to 0,02795 | No | ns | >0,9999 |
| TUG vs. Fsk 10 | -0,06558 | -0,1146 to -0,01658 | Yes | ** | 0,0026 |
| SST vs. SST- | -0,02945 | -0,04573 to -0,01317 | Yes | **** | <0,0001 |
| SST vs. TUG- | -0,03064 | -0,05575 to -0,005532 | Yes | ** | 0,0076 |
| SST vs. Fsk 10 | -0,09493 | -0,1419 to -0,04796 | Yes | **** | <0,0001 |
| SST- vs. TUG- | -0,001192 | -0,01605 to 0,01366 | No | ns | >0,9999 |
| SST- vs. Fsk 10 | -0,06548 | -0,09916 to -0,03180 | Yes | **** | <0,0001 |
| TUG- vs. Fsk 10 | -0,06429 | -0,09027 to -0,03831 | Yes | **** | <0,0001 |
|  |  |  |  |  |  |
| WT-cytosol |  |  |  |  |  |
| basal vs. TUG | -0,03280 | -0,05040 to -0,01520 | Yes | **** | <0,0001 |
| basal vs. SST | -0,01043 | -0,03273 to 0,01187 | No | ns | 0,9154 |
| basal vs. SST- | -0,06667 | -0,1042 to -0,02917 | Yes | **** | <0,0001 |
| basal vs. TUG- | -0,06875 | -0,1131 to -0,02440 | Yes | *** | 0,0003 |
| basal vs. Fsk 10 | -0,1864 | -0,2728 to -0,1001 | Yes | **** | <0,0001 |
| TUG vs. SST | 0,02237 | 0,009734 to 0,03501 | Yes | **** | <0,0001 |
| TUG vs. SST- | -0,03387 | -0,06241 to -0,005324 | Yes | * | 0,0100 |
| TUG vs. TUG- | -0,03595 | -0,07391 to 0,002011 | No | ns | 0,0766 |
| TUG vs. Fsk 10 | -0,1536 | -0,2320 to -0,07526 | Yes | **** | <0,0001 |
| SST vs. SST- | -0,05624 | -0,08476 to -0,02771 | Yes | **** | <0,0001 |
| SST vs. TUG- | -0,05832 | -0,09437 to -0,02227 | Yes | *** | 0,0002 |
| SST vs. Fsk 10 | -0,1760 | -0,2541 to -0,09786 | Yes | **** | <0,0001 |
| SST- vs. TUG- | -0,002084 | -0,01479 to 0,01063 | No | ns | >0,9999 |
| SST- vs. Fsk 10 | -0,1198 | -0,1765 to -0,06306 | Yes | **** | <0,0001 |
| TUG- vs. Fsk 10 | -0,1177 | -0,1727 to -0,06262 | Yes | **** | <0,0001 |

|  |  |  |  |  |  |
| --- | --- | --- | --- | --- | --- |
|  |  |  |  |  |  |
|  |  |  |  |  |  |
|  |  |  |  |  |  |
|  |  |  |  |  |  |
