# Supplementary figures and images for: Somatostatin triggers local cAMP and Ca2+ signaling in primary cilia to modulate pancreatic β-cell function (part 2 of 2)
Source: EMBO J. 2025 Feb 12;44(6):1663–91. doi: 10.1038/s44318-025-00383-7 (PMC11914567; doi:10.1038/s44318-025-00383-7)

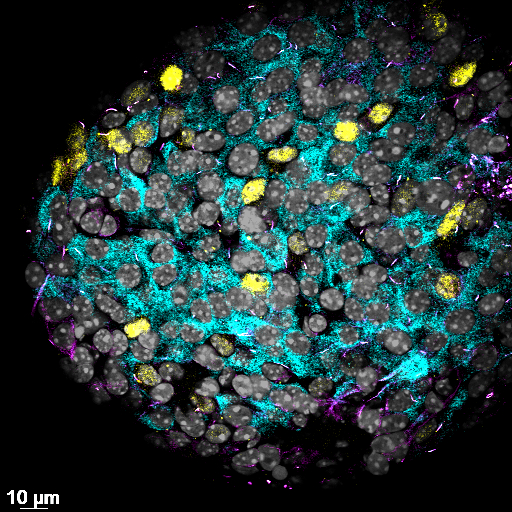

Supplement: Supplementary file 10 — Source data Fig. 8 [file 44318_2025_383_MOESM10_ESM.zip › Figure 8/8A and B/SAG/Composite (RGB).tif]

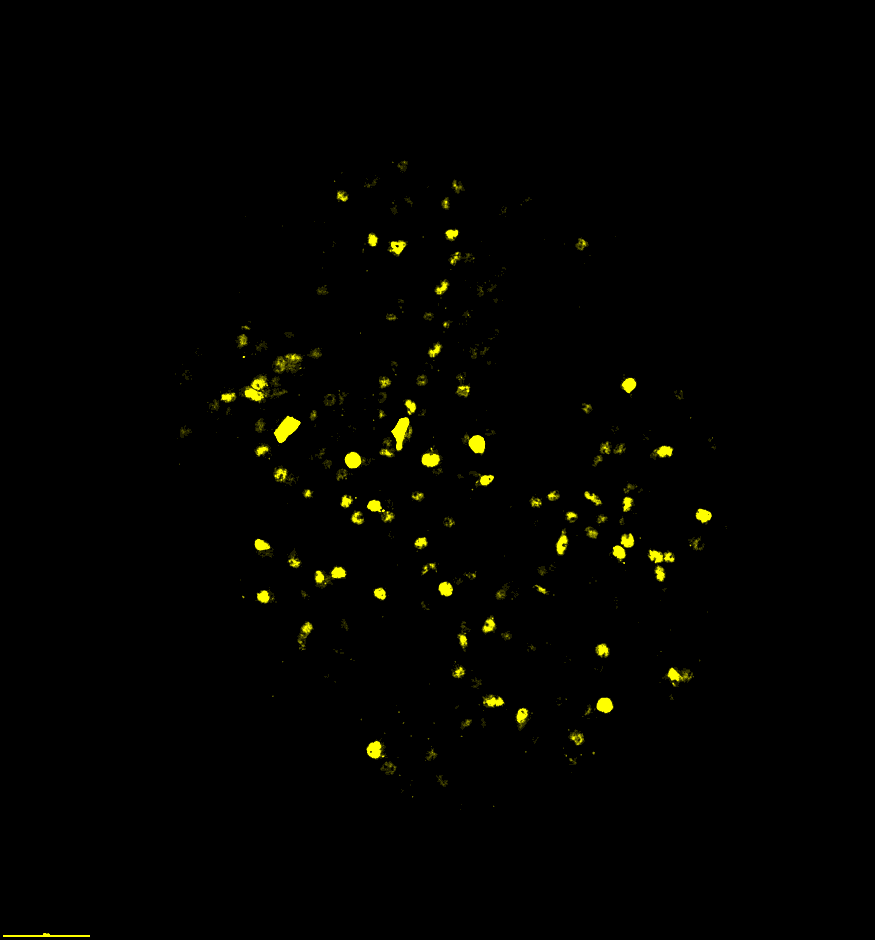

Supplement: Supplementary file 10 — Source data Fig. 8 [file 44318_2025_383_MOESM10_ESM.zip › Figure 8/8A and B/control/mouse islet Halo-Gli650 AcT488 Ins567 ctrl_Region 1_Merged_ch02_SV-1.tif]

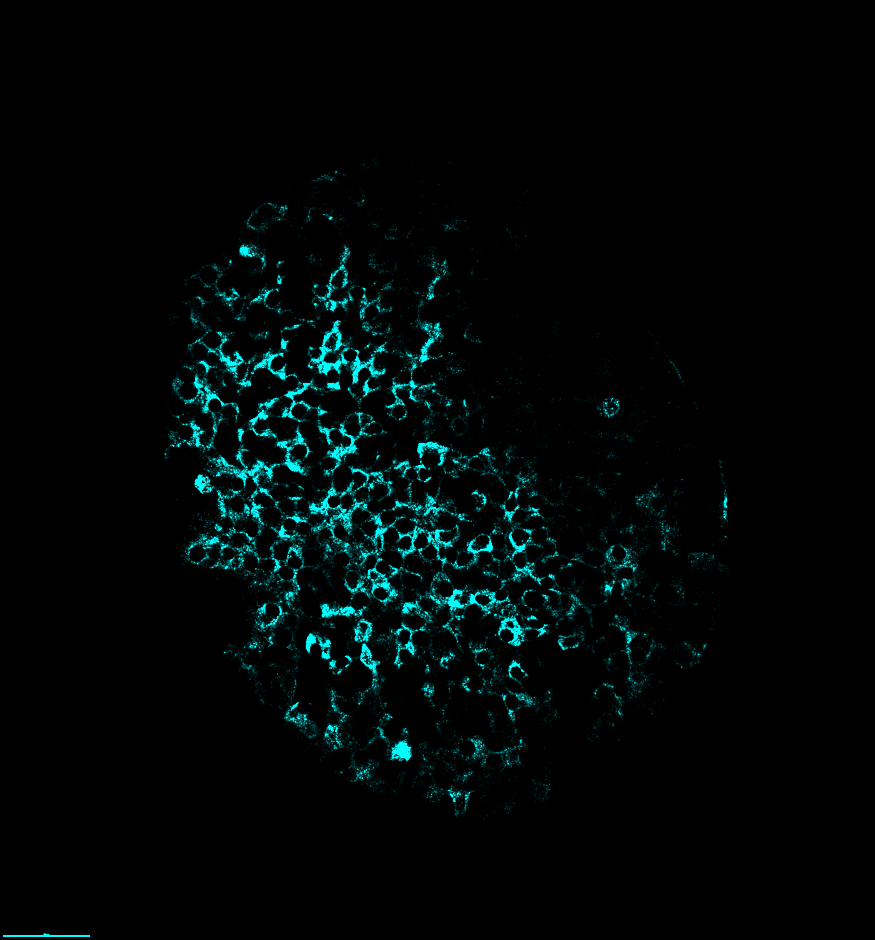

Supplement: Supplementary file 10 — Source data Fig. 8 [file 44318_2025_383_MOESM10_ESM.zip › Figure 8/8A and B/control/mouse islet Halo-Gli650 AcT488 Ins567 ctrl_Region 1_Merged_ch03_SV-1.tif]

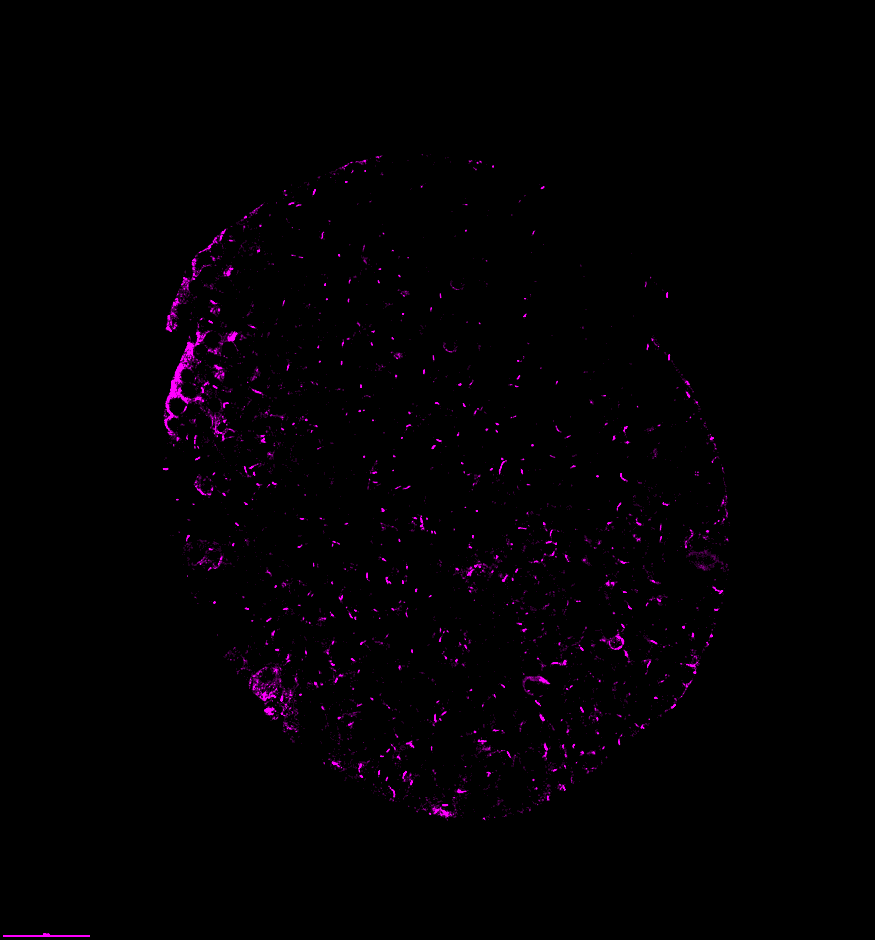

Supplement: Supplementary file 10 — Source data Fig. 8 [file 44318_2025_383_MOESM10_ESM.zip › Figure 8/8A and B/control/mouse islet Halo-Gli650 AcT488 Ins567 ctrl_Region 1_Merged_ch01_SV-1.tif]

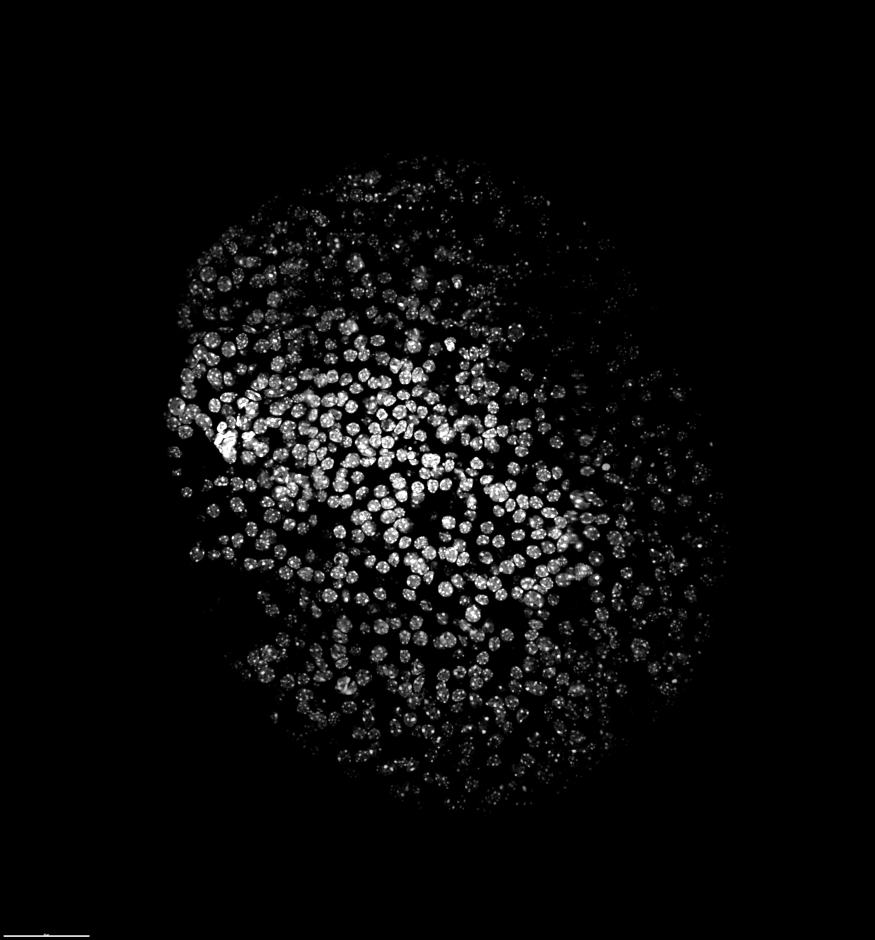

Supplement: Supplementary file 10 — Source data Fig. 8 [file 44318_2025_383_MOESM10_ESM.zip › Figure 8/8A and B/control/mouse islet Halo-Gli650 AcT488 Ins567 ctrl_Region 1_Merged_ch00_SV-1.tif]

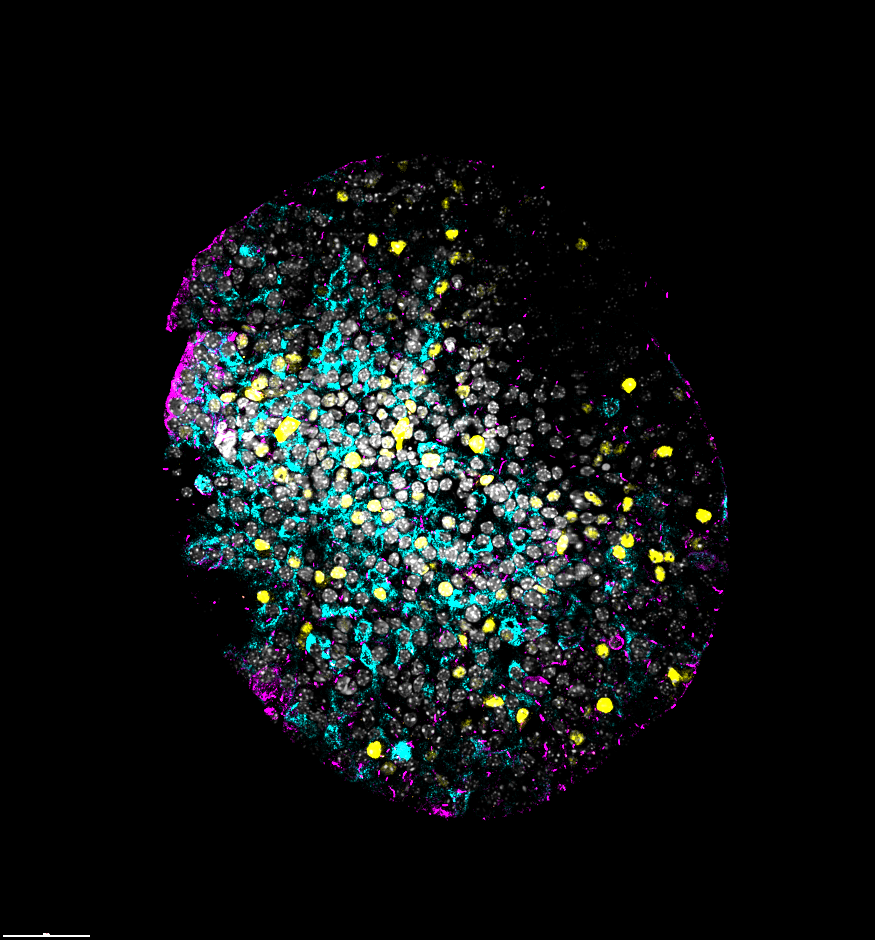

Supplement: Supplementary file 10 — Source data Fig. 8 [file 44318_2025_383_MOESM10_ESM.zip › Figure 8/8A and B/control/Composite (RGB).tif]

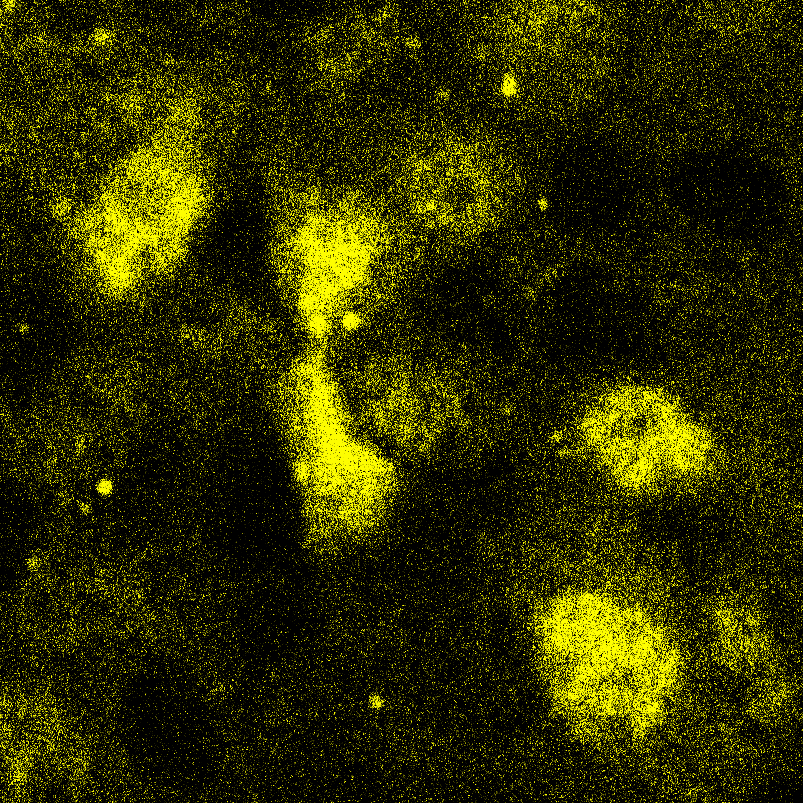

Supplement: Supplementary file 10 — Source data Fig. 8 [file 44318_2025_383_MOESM10_ESM.zip › Figure 8/8A and B/SST/zoom in/gli2.tif]

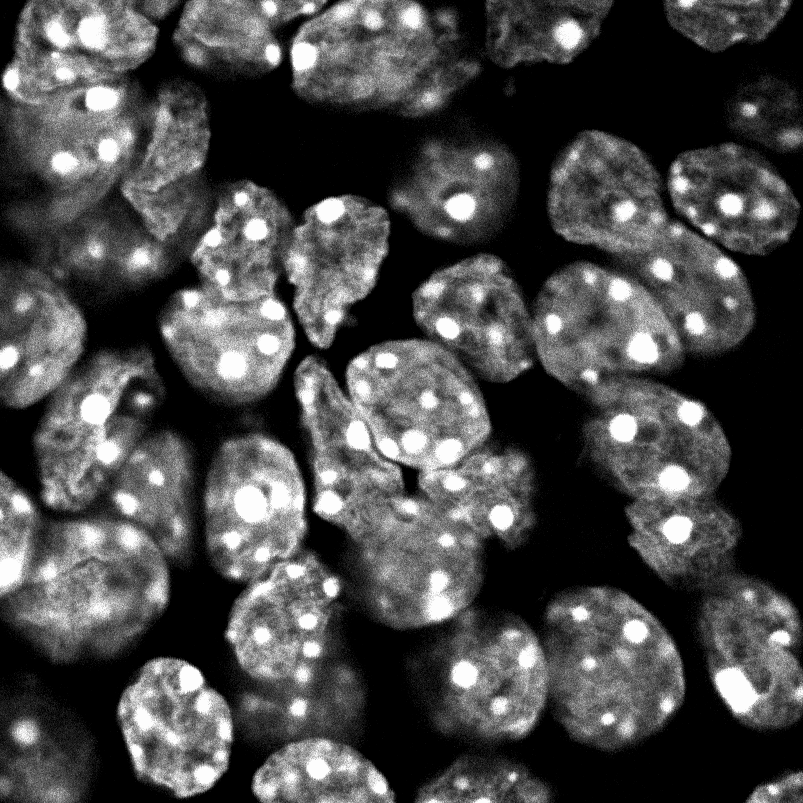

Supplement: Supplementary file 10 — Source data Fig. 8 [file 44318_2025_383_MOESM10_ESM.zip › Figure 8/8A and B/SST/zoom in/dapi.tif]

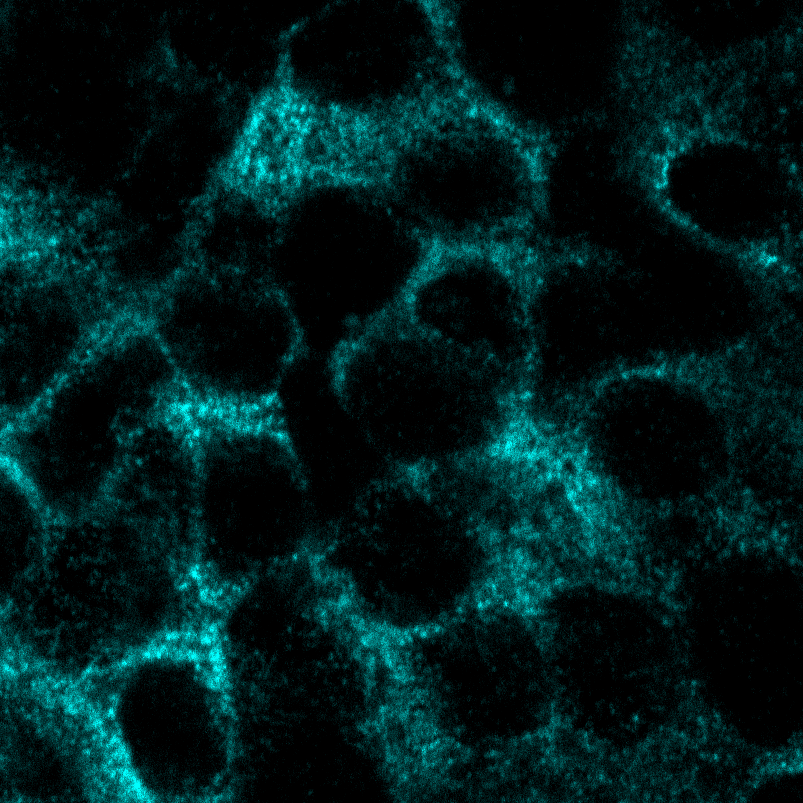

Supplement: Supplementary file 10 — Source data Fig. 8 [file 44318_2025_383_MOESM10_ESM.zip › Figure 8/8A and B/SST/zoom in/ins.tif]

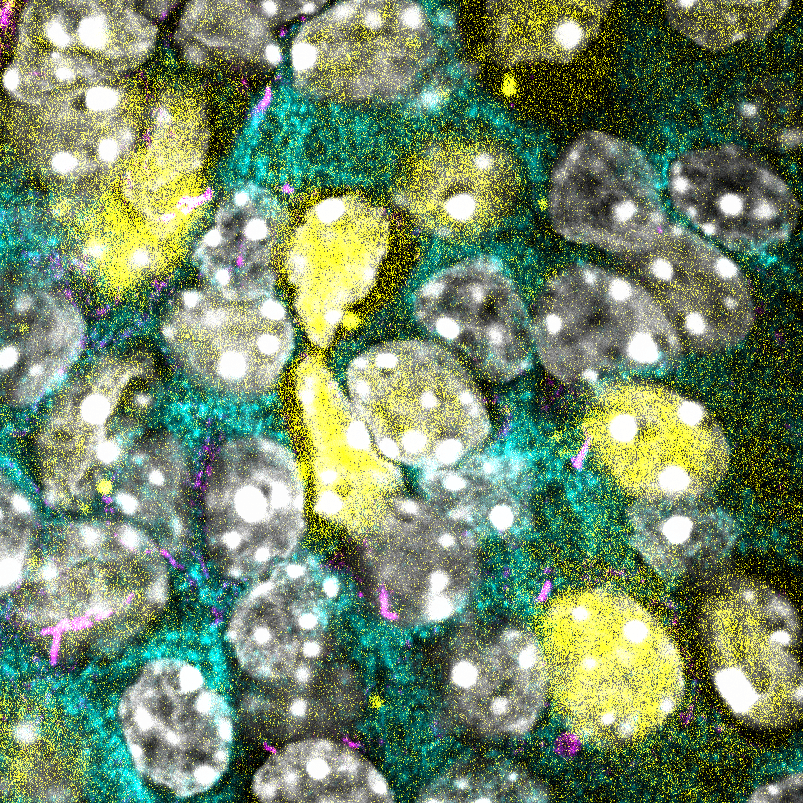

Supplement: Supplementary file 10 — Source data Fig. 8 [file 44318_2025_383_MOESM10_ESM.zip › Figure 8/8A and B/SST/zoom in/Composite (RGB).tif]

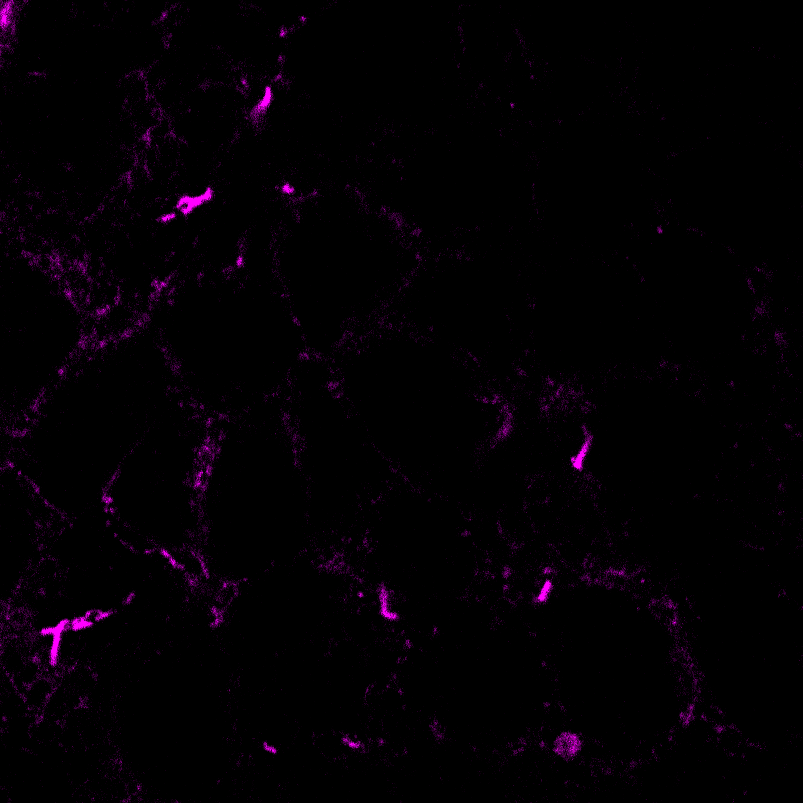

Supplement: Supplementary file 10 — Source data Fig. 8 [file 44318_2025_383_MOESM10_ESM.zip › Figure 8/8A and B/SST/zoom in/cilia.tif]

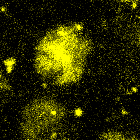

Supplement: Supplementary file 10 — Source data Fig. 8 [file 44318_2025_383_MOESM10_ESM.zip › Figure 8/8A and B/SST/inset/GLI2.tif]

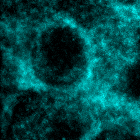

Supplement: Supplementary file 10 — Source data Fig. 8 [file 44318_2025_383_MOESM10_ESM.zip › Figure 8/8A and B/SST/inset/insulin.tif]

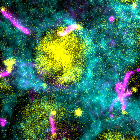

Supplement: Supplementary file 10 — Source data Fig. 8 [file 44318_2025_383_MOESM10_ESM.zip › Figure 8/8A and B/SST/inset/merge.tif]

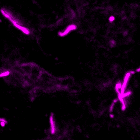

Supplement: Supplementary file 10 — Source data Fig. 8 [file 44318_2025_383_MOESM10_ESM.zip › Figure 8/8A and B/SST/inset/cilia.tif]

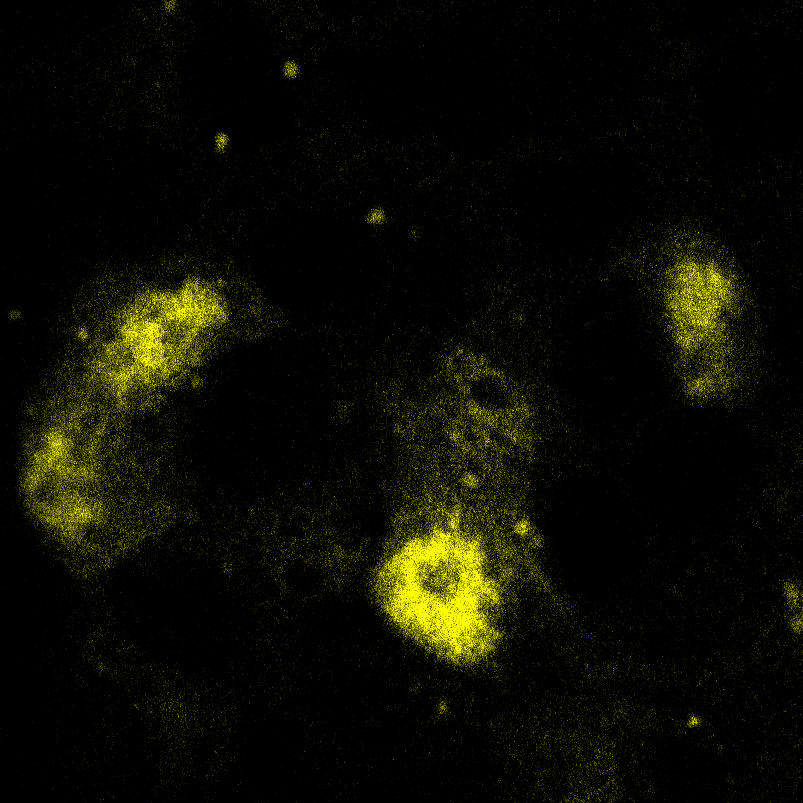

Supplement: Supplementary file 10 — Source data Fig. 8 [file 44318_2025_383_MOESM10_ESM.zip › Figure 8/8A and B/SAG/zoom in/gli2.tif]

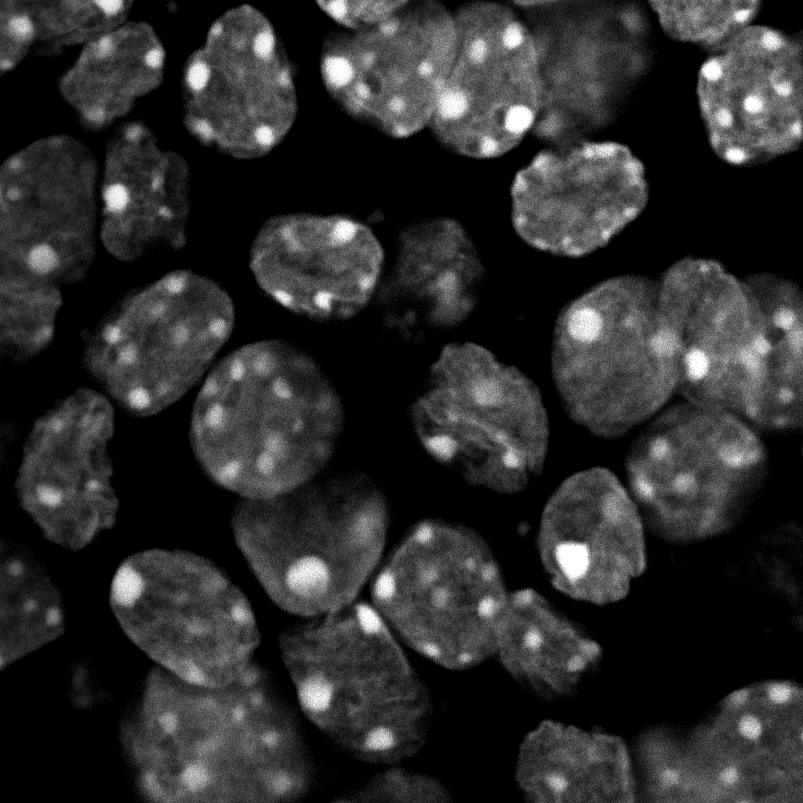

Supplement: Supplementary file 10 — Source data Fig. 8 [file 44318_2025_383_MOESM10_ESM.zip › Figure 8/8A and B/SAG/zoom in/dapi.tif]

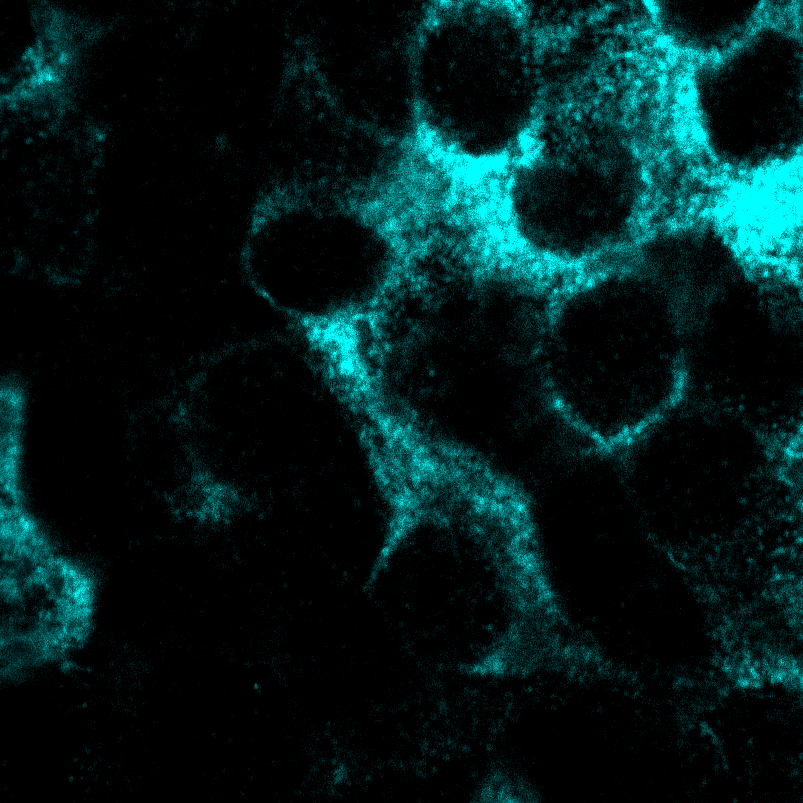

Supplement: Supplementary file 10 — Source data Fig. 8 [file 44318_2025_383_MOESM10_ESM.zip › Figure 8/8A and B/SAG/zoom in/ins.tif]

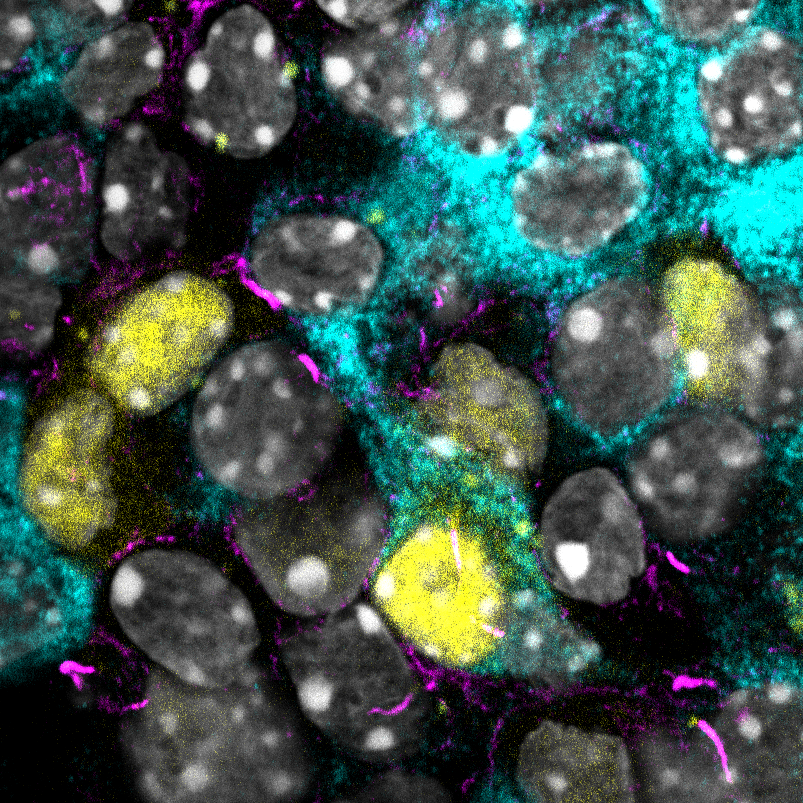

Supplement: Supplementary file 10 — Source data Fig. 8 [file 44318_2025_383_MOESM10_ESM.zip › Figure 8/8A and B/SAG/zoom in/Composite (RGB).tif]

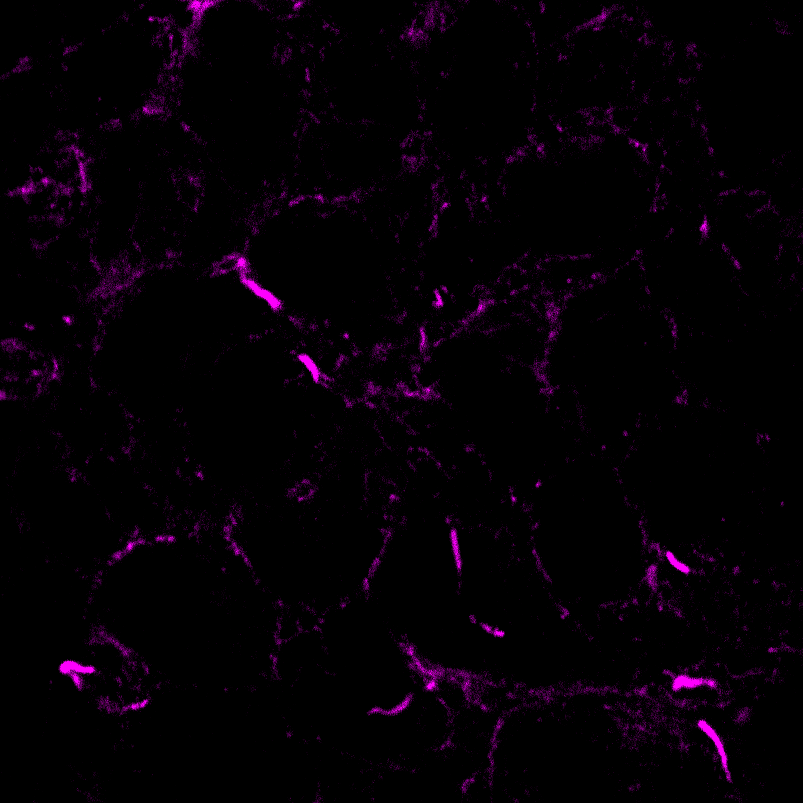

Supplement: Supplementary file 10 — Source data Fig. 8 [file 44318_2025_383_MOESM10_ESM.zip › Figure 8/8A and B/SAG/zoom in/cilia.tif]

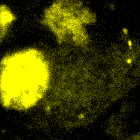

Supplement: Supplementary file 10 — Source data Fig. 8 [file 44318_2025_383_MOESM10_ESM.zip › Figure 8/8A and B/SAG/inset/GLI2.tif]

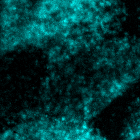

Supplement: Supplementary file 10 — Source data Fig. 8 [file 44318_2025_383_MOESM10_ESM.zip › Figure 8/8A and B/SAG/inset/insulin.tif]

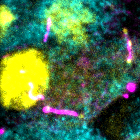

Supplement: Supplementary file 10 — Source data Fig. 8 [file 44318_2025_383_MOESM10_ESM.zip › Figure 8/8A and B/SAG/inset/merge.tif]

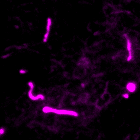

Supplement: Supplementary file 10 — Source data Fig. 8 [file 44318_2025_383_MOESM10_ESM.zip › Figure 8/8A and B/SAG/inset/cilia.tif]

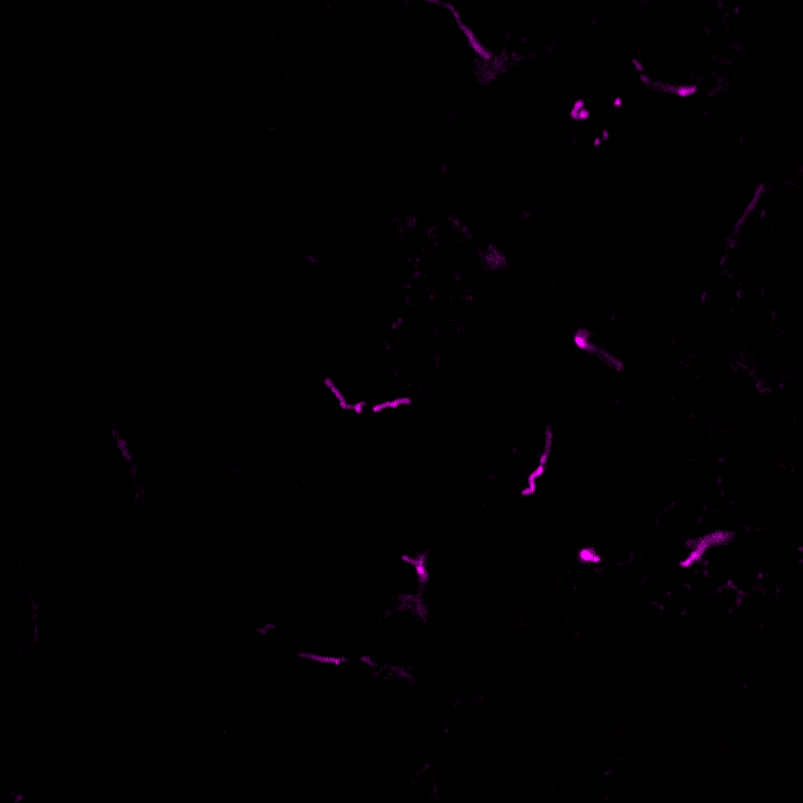

Supplement: Supplementary file 10 — Source data Fig. 8 [file 44318_2025_383_MOESM10_ESM.zip › Figure 8/8A and B/control/zoom in/actub.tif]

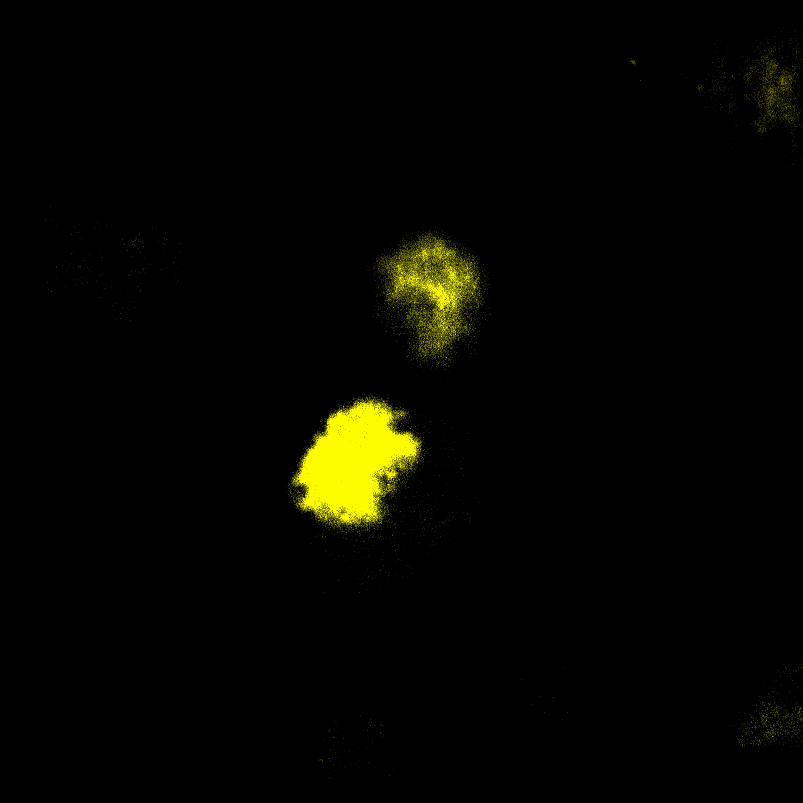

Supplement: Supplementary file 10 — Source data Fig. 8 [file 44318_2025_383_MOESM10_ESM.zip › Figure 8/8A and B/control/zoom in/gli2.tif]

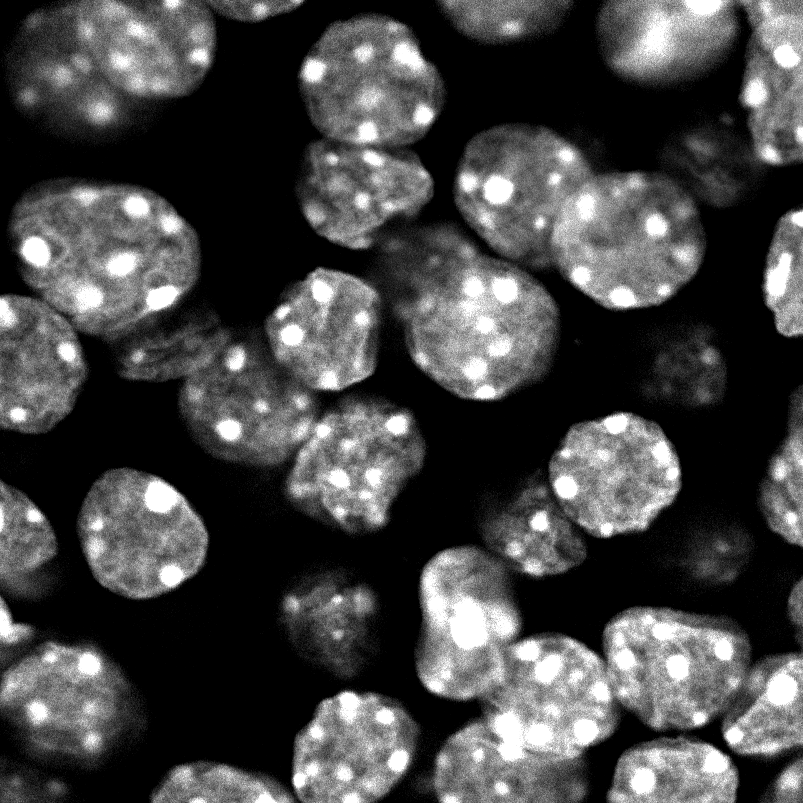

Supplement: Supplementary file 10 — Source data Fig. 8 [file 44318_2025_383_MOESM10_ESM.zip › Figure 8/8A and B/control/zoom in/dapi.tif]

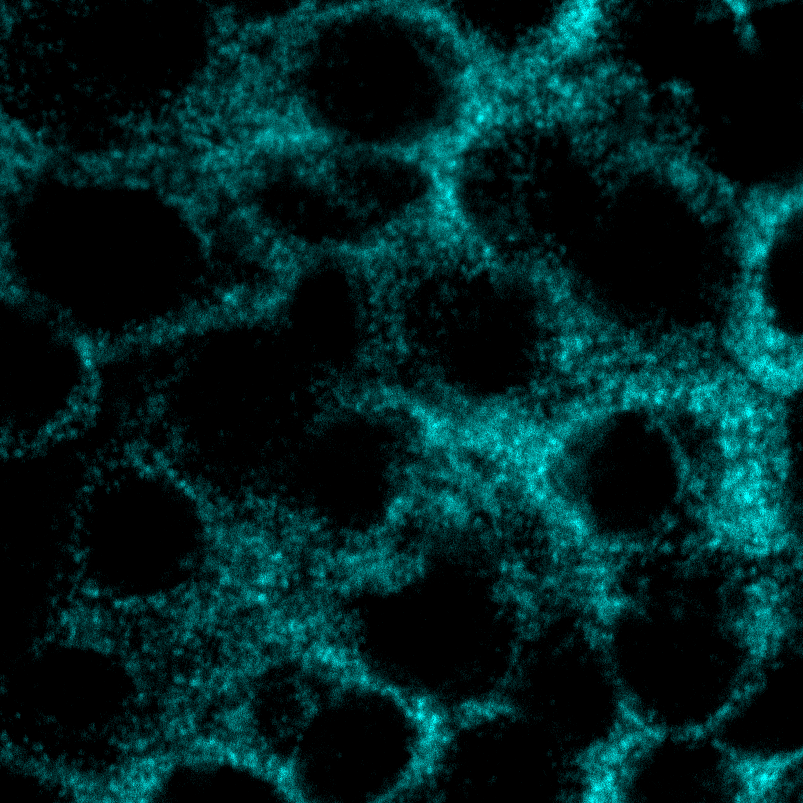

Supplement: Supplementary file 10 — Source data Fig. 8 [file 44318_2025_383_MOESM10_ESM.zip › Figure 8/8A and B/control/zoom in/ins.tif]

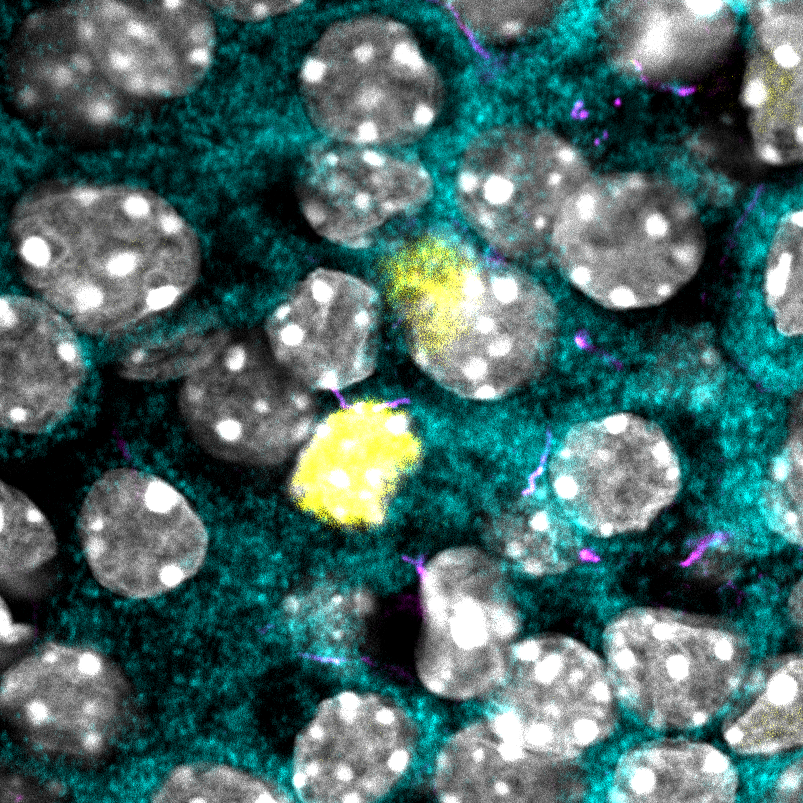

Supplement: Supplementary file 10 — Source data Fig. 8 [file 44318_2025_383_MOESM10_ESM.zip › Figure 8/8A and B/control/zoom in/Composite (RGB).tif]

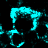

Supplement: Supplementary file 10 — Source data Fig. 8 [file 44318_2025_383_MOESM10_ESM.zip › Figure 8/8A and B/control/inset/mouse islet Halo-Gli650 AcT488 Ins567 ctrl_Region 1_Merged_ch03_SV-2.tif]

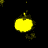

Supplement: Supplementary file 10 — Source data Fig. 8 [file 44318_2025_383_MOESM10_ESM.zip › Figure 8/8A and B/control/inset/mouse islet Halo-Gli650 AcT488 Ins567 ctrl_Region 1_Merged_ch02_SV-2.tif]

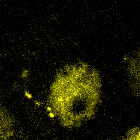

Supplement: Supplementary file 10 — Source data Fig. 8 [file 44318_2025_383_MOESM10_ESM.zip › Figure 8/8A and B/control/inset/GLI2.tif]

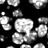

Supplement: Supplementary file 10 — Source data Fig. 8 [file 44318_2025_383_MOESM10_ESM.zip › Figure 8/8A and B/control/inset/mouse islet Halo-Gli650 AcT488 Ins567 ctrl_Region 1_Merged_ch00_SV-2.tif]

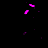

Supplement: Supplementary file 10 — Source data Fig. 8 [file 44318_2025_383_MOESM10_ESM.zip › Figure 8/8A and B/control/inset/mouse islet Halo-Gli650 AcT488 Ins567 ctrl_Region 1_Merged_ch01_SV-2.tif]

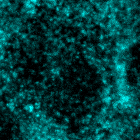

Supplement: Supplementary file 10 — Source data Fig. 8 [file 44318_2025_383_MOESM10_ESM.zip › Figure 8/8A and B/control/inset/insulin.tif]

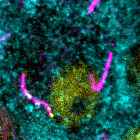

Supplement: Supplementary file 10 — Source data Fig. 8 [file 44318_2025_383_MOESM10_ESM.zip › Figure 8/8A and B/control/inset/merge.tif]

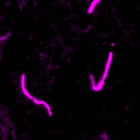

Supplement: Supplementary file 10 — Source data Fig. 8 [file 44318_2025_383_MOESM10_ESM.zip › Figure 8/8A and B/control/inset/cilia.tif]
