# Supplementary material for: Somatostatin triggers local cAMP and Ca2+ signaling in primary cilia to modulate pancreatic β-cell function
Source: EMBO J. 2025 Feb 12;44(6):1663–91. doi: 10.1038/s44318-025-00383-7 (PMC11914567; doi:10.1038/s44318-025-00383-7)
Supplement: Supplementary file 12 — Expanded View Figures [file 44318_2025_383_MOESM12_ESM.pdf]

Expanded View Figures

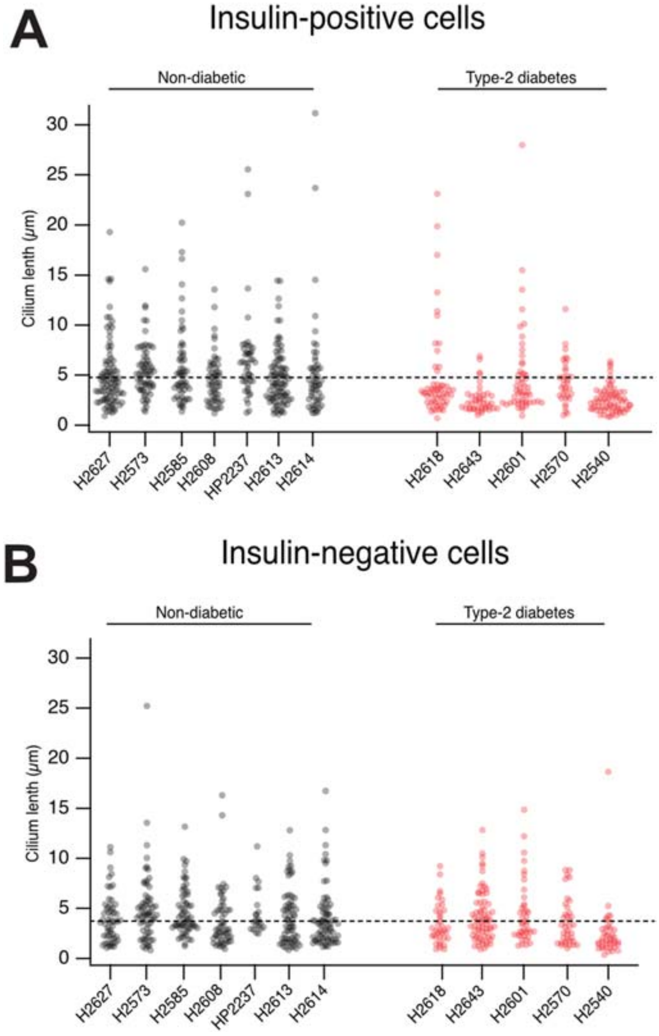

Figure EV1. Cilia length in human islets from non-diabetic and type-2 diabetic donors.

(**A**) Quantification of cilium length in insulin-positive β-cells in islets from 7 non-diabetic (black; $n = 94$, 66, 57, 56, 45, 92 and 54 cilia) and 5 type-2 diabetic (red; $n = 57$, 42, 53, 33 and 71 cilia) human organ donors. Dashed line shows average for all non-diabetic donors. (**B**) Quantification of cilium length in insulin-negative cells in islets from 7 non-diabetic (black; $n = 54$, 65, 63, 54, 22, 66 and 74 cilia) and 5 type-2 diabetic (red; $n = 37$, 70, 40, 38 and 40 cilia) human organ donors. Dashed line shows average for all non-diabetic donors. Source data are available online for this figure.

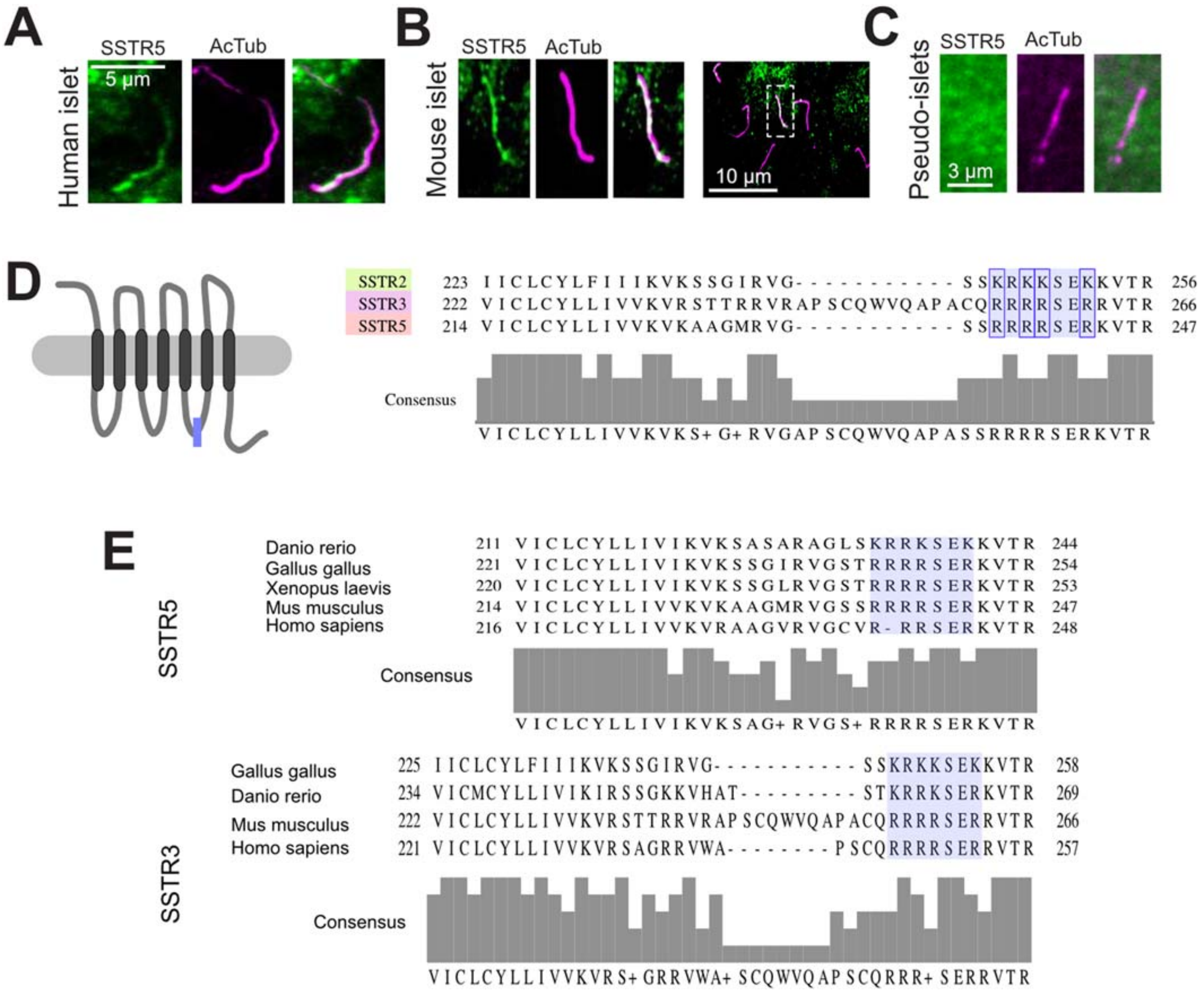

Figure EV2. SSTR5 localized to primary cilia.

(**A**) Confocal microscopy image of a primary cilium (acetylated tubulin; magenta) in a human islet that expresses SSTR5 (green). (**B**) Confocal microscopy image of primary cilia (acetylated tubulin; magenta) in a mouse islet that expresses SSTR5 (green). (**C**) Confocal microscopy image of a primary cilium (acetylated tubulin; magenta) in a MIN6 pseudoislet that lack expression of SSTR5 (green). (**D**) An illustration shows the membrane localization of SSTRs, with IC3 loop indicated by the purple box. Sequences of mSSTR2, mSSTR3 and mSSTR5. Conserved motif RxRxxR is highlighted. (**E**) Sequences of SSTR5 and SSTR3 showing the evolutionary conservation of a stretch of amino acids in purple. Source data are available online for this figure.

 © The Author(s)

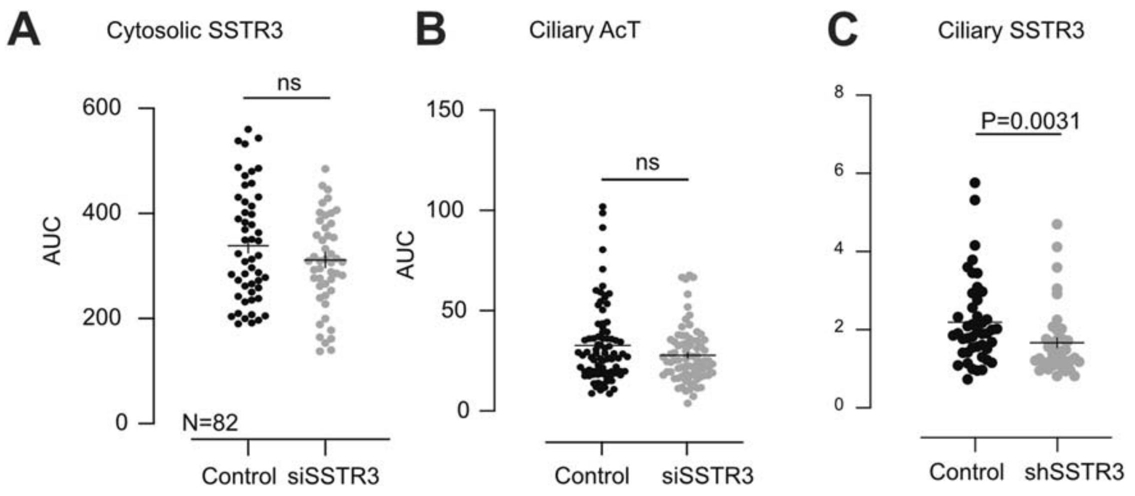

Figure EV3. SSTR3 is a ciliary somatostatin receptor.

(**A**) Quantification of cytosolic area selected in MIN6 pseudoislets. Fluorescence intensity is from control (black) and SSTR3 KD (gray) cells immunostained for SSTR3. (means ± SEM; $n_{ctrl} = 50$ and $n_{KD} = 48$, 3 different preparations, no statistical difference by Mann–Whitney *U* test, unpaired.). (**B**) Quantifications of line profiles drawn along cilia of MIN6 pseudoislets for acetylated tubulin. Acetylated tubulin signal is unaffected on the left. (means ± SEM; $n_{ctrl} = 83$ and $n_{KD} = 86$, three different preparations, no statistical difference by Mann–hitney *U* test, unpaired.). (**C**) Quantifications of line profiles drawn along cilia of MIN6 pseudoislets positive for SSTR3. Ciliary SSTR3 signal is significantly reduced in shSSTR3 expressing cells (means ± SEM; $n_{ctrl} = 44$ and $n_{KD} = 40$ cilia, 1 preparation; Mann–Whitney *U* test, unpaired). Source data are available online for this figure.

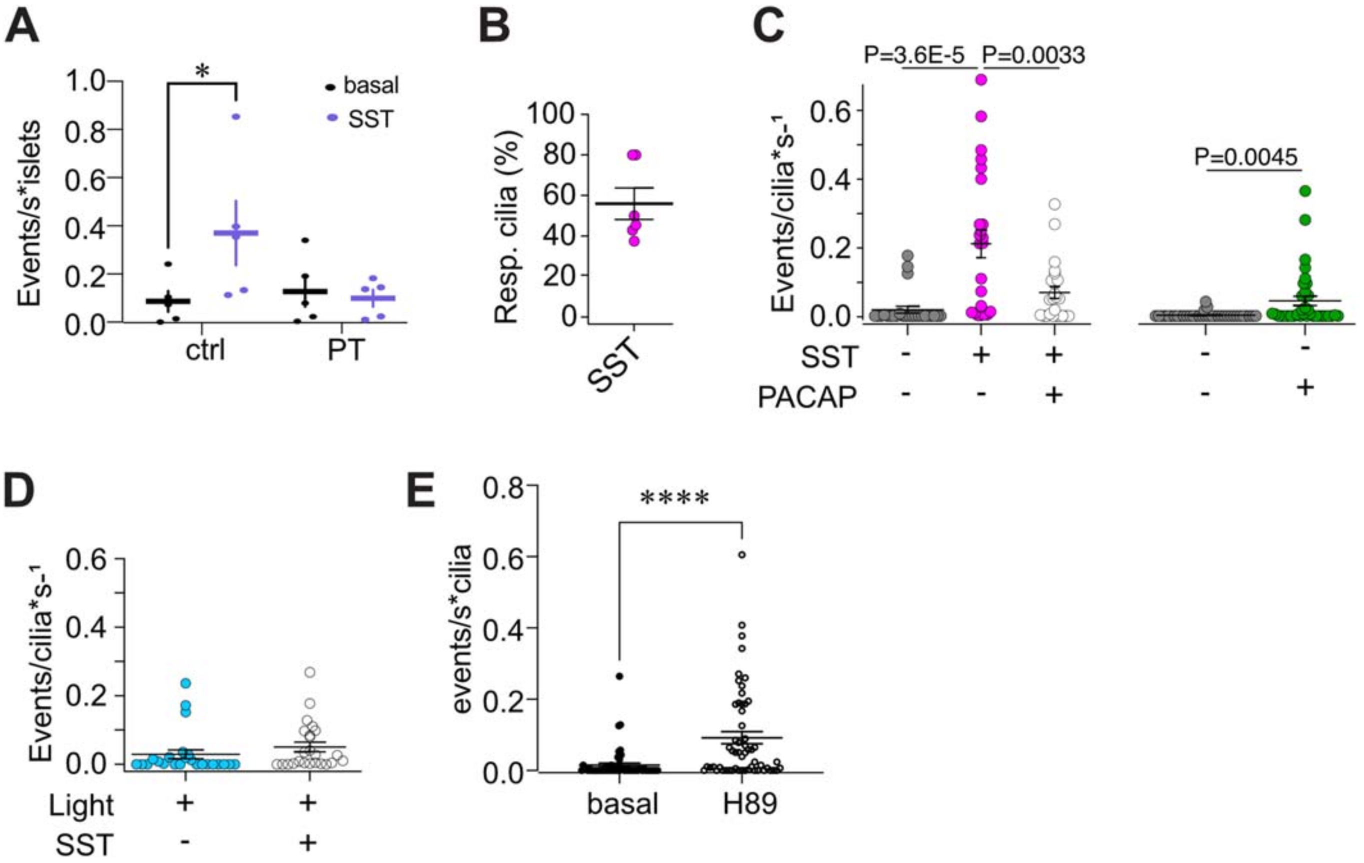

Figure EV4. Elevation of cAMP counteracts somatostatin-induced ciliary Ca^{2+} signaling.

(**A**) MIN6 pseudoislets were cultured under control condition (black) or in the presence of pertussis toxin (PT; purple) for 18 h. Quantifications of the Ca^{2+} responses to 100 nM somatostatin are shown to the right (means ± SEM; $n_{ctrl} = 5$ islets, $n_{PT} = 5$ islets; from three different preparations, SST response in control $P = 0.0425$, SST response in PT not significant, assessed by Sidak´s multiple comparison). (**B**) Fraction of MIN6 cell cilia per pseudoislet that exhibit somatostatin-induced ciliary Ca^{2+} signaling (means ± SEM; $n = 6$ islets). (**C**) Ciliary Ca^{2+} responses under basal conditions (gray) or in the presence of 100 nM somatostatin (magenta), a combination of somatostatin and 100 nM PACAP (white) or 100 nM PACAP alone (green) in MIN6 pseudoislets (means ± SEM; left panel: $n = 25$ cilia from 6 islets; right panel: $n = 36$ cilia from 4 islets; Student's paired t test). (**D**) Ciliary Ca^{2+} responses to 100 nM somatostatin in MIN6 pseudoislets co-expressing cytosolic bPac. bPac activity was continuously stimulated during the experiment by 491-nm illumination (Light). (mean s± SEM; $n = 24$ cilia from 3 islets; Student's paired t test). (**E**) Event count of ciliary Ca^{2+} changes from mouse islets in response to 10 μM H89 (means ± SEM; 7 islets; 56 cilia; 3 different preparations. ****$P < 0.0001$, Wilcoxon-matched pair t test). Source data are available online for this figure.

 © The Author(s)

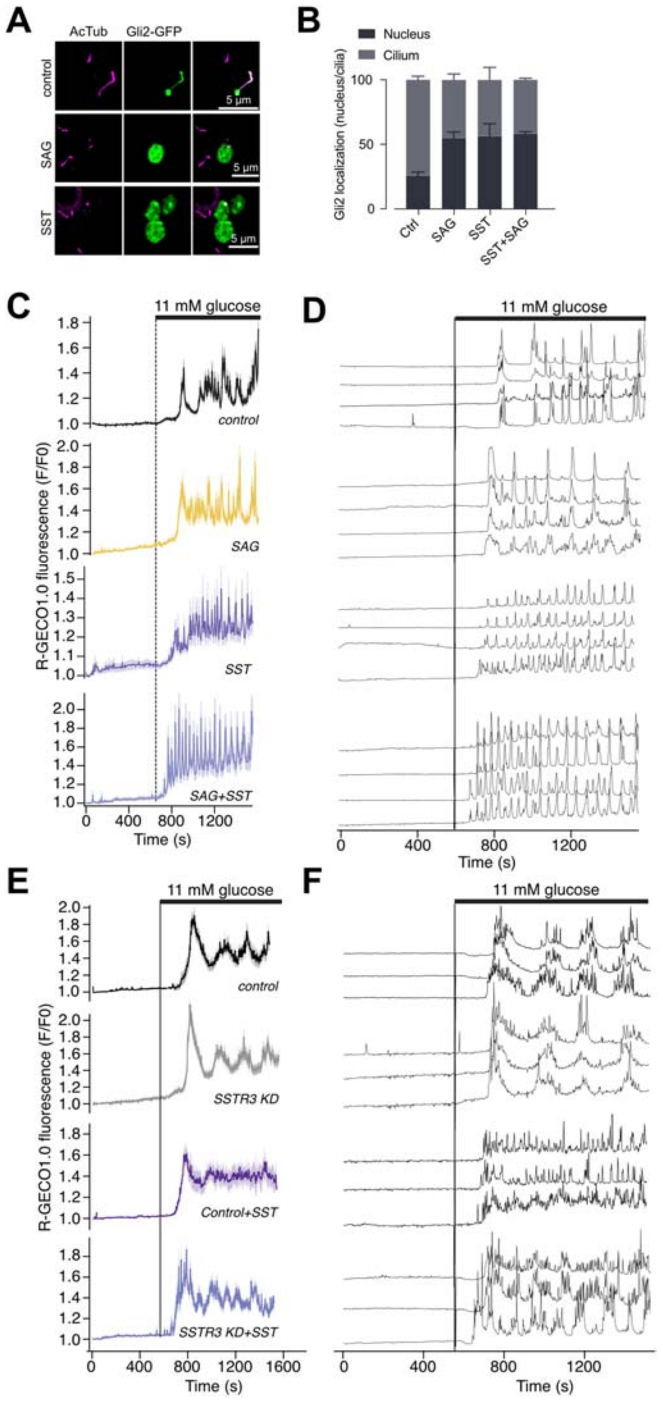

Figure EV5. Long-term somatostatin stimulation alters beta cell Ca^{2+} response to glucose.

(**A**) Confocal microscopy images of MIN6 cells expressing Gli2-GFP (green) and immunostained against acetylated tubulin (magenta). 18 h treatment with 100 nM SAG and 100 nM SST induced translocation of Gli2 to the nucleus. (**B**) Means ± SEM for the nucleus/cilia Gli2 ratio change in MIN6 cells ($P_{SAG} = 0.0345$, $P_{SST} = 0.0266$, $P_{SAG+SST} = 0.0186$ all compared to control and assessed by Sidak´s multiple comparison test). (**C**, **D**) Islet averages (**C**) and example recordings (**D**) of glucose-induced R-GECO1 fluorescence changes in MIN6 pseudoislets treated for 18 h with DMSO (control), 100 nM SAG, 100 nM somatostatin or 100 nM SAG in combination with 100 nM somatostatin ($n = 3$ replicates). (**E**, **F**) Islet averages (**E**) and example recordings (**F**) of glucose-induced R-GECO1 fluorescence changes in control and SSTR3 knockdown MIN6 pseudoislets treated for 18 h with DMSO (control) or 100 nM somatostatin ($n = 3$ replicates). Source data are available online for this figure.

© The Author(s)
